# Localized Interleukin-12 for Cancer Immunotherapy

**DOI:** 10.3389/fimmu.2020.575597

**Published:** 2020-10-15

**Authors:** Khue G. Nguyen, Maura R. Vrabel, Siena M. Mantooth, Jared J. Hopkins, Ethan S. Wagner, Taylor A. Gabaldon, David A. Zaharoff

**Affiliations:** Joint Department of Biomedical Engineering, University of North Carolina, Chapel Hill and North Carolina State University, Raleigh, NC, United States

**Keywords:** interleukin-12 (IL-12), cancer immunotherapy, cancer vaccine, cytokine delivery system, localized delivery, intratumoral administration

## Abstract

Interleukin-12 (IL-12) is a potent, pro-inflammatory type 1 cytokine that has long been studied as a potential immunotherapy for cancer. Unfortunately, IL-12's remarkable antitumor efficacy in preclinical models has yet to be replicated in humans. Early clinical trials in the mid-1990's showed that systemic delivery of IL-12 incurred dose-limiting toxicities. Nevertheless, IL-12's pleiotropic activity, i.e., its ability to engage multiple effector mechanisms and reverse tumor-induced immunosuppression, continues to entice cancer researchers. The development of strategies which maximize IL-12 delivery to the tumor microenvironment while minimizing systemic exposure are of increasing interest. Diverse IL-12 delivery systems, from immunocytokine fusions to polymeric nanoparticles, have demonstrated robust antitumor immunity with reduced adverse events in preclinical studies. Several localized IL-12 delivery approaches have recently reached the clinical stage with several more at the precipice of translation. Taken together, localized delivery systems are supporting an IL-12 renaissance which may finally allow this potent cytokine to fulfill its considerable clinical potential. This review begins with a brief historical account of cytokine monotherapies and describes how IL-12 went from promising new cure to ostracized black sheep following multiple on-study deaths. The bulk of this comprehensive review focuses on developments in diverse localized delivery strategies for IL-12-based cancer immunotherapies. Advantages and limitations of different delivery technologies are highlighted. Finally, perspectives on how IL-12-based immunotherapies may be utilized for widespread clinical application in the very near future are offered.

## Overview of IL-12-Based Immunotherapies

### A Brief History of Cytokine and IL-12 Immunotherapies

Since the discovery of an “endogenous pyrogen,” now known as IL-1, in 1953, scientists have anticipated the use of exogenous cytokines to manipulate a patient's immune system in an effort to control malignant neoplasms (1). Early obstacles to cytokine-based immunotherapy centered on difficulties achieving reproducible manufacture of a sufficient and pure supply of cytokines for clinical trials. In the early 1980s, recombinant DNA technology and advances in the biochemical characterization of proteins combined to overcome this hurdle. Finally, in 1986, IFN-α broke through as the first cytokine to win FDA approval as a single agent cytokine therapy for cancer (2). Since then, hundreds, if not thousands, of studies have evaluated more than 40 cytokines against a range of preclinical tumor models. A number of promising cytokines, including GM-CSF, IL-1, TNFα, IFN-γ, and IL-12, subsequently entered clinical trials as single agents but failed to provide clinical benefit. Currently, only 2 of 40+ identified cytokines are approved as single agent immunotherapies for a limited number of indications (Table 1). Another FDA approved cancer immunotherapy, Talimogene laherparepvec (T-VEC; Imlygic™) is an oncolytic herpes simplex virus that uses GM-CSF expression as an immune enhancer (7).

**Table 1:** Approved cytokine monotherapies and cytokine-enhanced therapies for cancer.

**Cytokine**	**Approved indications**	**Route of administration**	**References**
IFN-α (2a or 2b)	Hairy cell leukemia; Chronic myelogenous leukemia; AIDS-related Kaposi's sarcoma	Intravenous injection Subcutaneous injection^*^ Intramuscular injection^*^	(3)
IL-2	Metastatic renal cell carcinoma; Metastatic melanoma	Intravenous injection	(4, 5)
Talimogene laherparepvec (T-VEC)	Unresectable advanced melanoma	Intratumoral injection	(6)

* *Subcutaneous and intramuscular injections are local deliveries. However, they result in systemic cytokine distribution and are utilized in the clinic as systemic therapies*.

Perhaps the greatest disappointment in cytokine immunotherapy development thus far is IL-12. IL-12 is a potent, pro-inflammatory cytokine produced by antigen presenting cells typically in response to microbial pathogens. It is comprised of two subunits, p35 and p40, that are linked by three disulfide bridges to form a p70 heterodimer (7–10). IL-12 is chiefly responsible for the induction and enhancement of cell-mediated immunity. Among its diverse functions, IL-12 has been shown to: (i) induce T_H_1 cell differentiation; (ii) increase activation and cytotoxic capacities of T and NK cells; and (iii) inhibit or reprogram immunosuppressive cells, such as tumor associated macrophages (TAMs) and myeloid-derived suppressor cells (MDSCs) (11–16). IL-12 also induces the production of large amounts of IFNγ which itself is cytostatic/cytotoxic (17, 18), anti-angiogenic (19, 20) and can upregulate MHC I and II expression on tumor cells for enhanced recognition and lysis (21). Not surprisingly then, IL-12 has demonstrated remarkable antitumor effects against a range of malignancies in preclinical studies (22–25). These effects are largely dependent on CD8^+^ T cells, NK cells, and NK T cells (23, 26, 27).

In clinical studies, IL-12 has been evaluated as an experimental treatment for numerous malignancies (28–38). Unfortunately, the efficacy of IL-12 at tolerated doses has been minimal (29, 30, 33). Atkins and colleagues were the first to employ IL-12 immunotherapy in a clinical trial (28). This phase I study enrolled 40 patients, including 20 with renal cancer and 12 with melanoma, to investigate intravenous administration of recombinant hIL-12 (rhIL-12). One melanoma patient experienced a transient complete response and one renal cancer patient had a partial response (28). Subcutaneous rhIL-12 was employed in a separate pilot study that enrolled 10 advanced melanoma patients (29). In this study, a fixed dose of rhIL-12 (0.5 μg/kg) was given to patients on days 1, 8, and 15 for two sequential cycles of 28 days. No partial or complete responses were reported. Minor tumor shrinkages involving some subcutaneous metastases and hepatic metastases were observed (29). In yet another early melanoma study, the administration of IL-12 was found to induce a striking peripheral burst of HLA-restricted CTL precursors directed to autologous tumors and to multiple immunogenic tumor-associated antigens (39). Significantly, the infiltration of CD8^+^ T cells with an effector-memory phenotype was identified in posttreatment metastatic lesions, but not in pretreatment metastatic lesions of three patients (39). IL-12 has also been shown to induce productive antitumor responses against cutaneous T cell lymphoma variants (38), AIDS-related Kaposi sarcoma (37), and non–Hodgkin's lymphoma (38).

Although IL-12 has demonstrated robust antitumor activity in preclinical studies and potent immune-stimulating potential in humans, systemic administrations of IL-12 have been shown to be exceedingly toxic. In one phase II trial, a maximal dose of 0.5 μg/kg per day resulted in severe side effects in 12 out of 17 enrolled patients and the deaths of two patients (40). Interestingly, the same dose of 0.5 μg/kg IL-12 per day was found to be well-tolerated in patients that were enrolled in a previous phase I study. A change in dosing schedule accounted for the differences in toxicity between the phase I and phase II trials. In the phase I trial, a single tester dose of IL-12 was administered 1 week before a multiple-dose regimen. The tester dose was found to blunt the toxicity induced by subsequent doses (41). Overall, severe toxicities in early clinical trials, including 2 on-study deaths (42) due to frequent systemic injections of IL-12, together with disappointing clinical responses in large phase 2 studies (43, 44), dampened enthusiasm for IL-12-based immunotherapy.

The disappointing antitumor responses in clinical trials raised the possibility that IL-12 is simply less active in humans. However, the severe toxicities outlined above indicate that IL-12 has potent biological activity in humans. Another possibility for the limited clinical efficacy is insufficient delivery of IL-12 to the tumor microenvironment in humans. IL-12, like most cytokines, functions locally through paracrine and autocrine mechanisms. The ideal targets of IL-12 immunotherapy are not lymphocytes in circulation, but rather immune cells within the tumor and nearby lymph nodes, including activated but exhausted T cells, NK cells, TAMs, and MDSCs. Therefore, maximizing the amount of IL-12 that reaches the tumor seems critical for a robust antitumor response.

### Justification for Localized IL-12 Delivery

We and others have noted that IL-12 immunotherapeutics would be more effective and less toxic if delivered and maintained in the tumor through the use of novel delivery technologies. There are five benefits of local, persistent IL-12 delivery. The first is enhanced spatiotemporal distribution of IL-12 compared to systemic delivery. The failure of IL-12-based immunotherapies to achieve widespread clinical success may be at least partially attributed to the inability of a tolerated dose of systemically administered IL-12 to reach therapeutic concentrations within human tumors. In mice, implanted or induced tumors are disproportionately large. For example, a 1.5-g tumor (~1.25 cm^3^) comprises 6% of the body weight of a 25-g mouse. A comparably sized tumor in a 70 kg (154 lbs) human would weigh 4.2 kg (9.2 lbs). Furthermore, rapidly growing murine tumors are highly vascularized relative to their human counterparts (45, 46). Taken together, a significantly larger fraction of a systemically administered IL-12 dose can be expected to reach the tumor in a mouse compared to a human. Localized delivery strategies, on the other hand, are capable of enhancing IL-12 concentrations in the tumor microenvironment by one or more orders of magnitude (47–50).

A second benefit of localized IL-12 delivery is the ability to generate systemic antitumor immunity from a locally initiated immune response. As cancer metastasizes and becomes a “systemic” disease, conventional wisdom has suggested that metastases must be treated with systemic therapies such as i.v. administered chemotherapies or immune checkpoint inhibitors. However, systemic delivery increases the frequency of adverse events through off-target interactions. For instance, systemic IL-12 therapy has the potential to cause activation and/or differentiation of all circulating T cells whereas activation/differentiation of only tumor-specific or tumor antigen-experienced T cells is preferred. Fortunately, a growing mountain of evidence demonstrates that localized IL-12 can generate systemic, adaptive immunological memory capable of controlling anestic tumors, inhibiting metastases, and preventing tumor recurrences (51–53). In particular, local administration of IL-12 has been shown to activate or reactivate tumor infiltrating CD8^+^ T cells, improve antigen presenting machinery and subsequently cause the expansion of tumor-specific CD8^+^ effector T cells. This often leads to enhanced infiltration of contralateral untreated tumors (54). Research from our own lab demonstrated that local/intravesical administration of IL-12 eliminated untreated flank tumors only when a primary orthotopic bladder tumor was treated. These data indicated that T cells must be educated with antigens from a primary tumor in order to find and eliminate abscopal tumors. Similarly, intratumoral injections of IL-12 neoadjuvant to resection have been shown to inhibit metastases by multiple groups in a T cell, NK cell, or IFN-γ dependent manner (52, 55). Taken together, the convincing evidence demonstrating that localized IL-12 can induce abscopal immunity renders systemic IL-12 delivery unnecessary even for the treatment of metastatic disease.

Third, as mentioned above, IL-12 is a pleiotropic cytokine with context dependent consequences. When IL-12 is administered systemically, it induces rapid increases in pro-inflammatory cytokines, such as IFN-γ, TNF-α, and IL-6 (56). This “cytokine storm” combined with rapid decreases in peripheral blood lymphocytes, monocytes, and neutrophils can be lethal (42). However, when controlled locally, pleiotropic cytokines have the potential to engage multiple antitumor effector mechanisms. For instance, IL-12 increases the activation and cytolytic capacity of CD8^+^ T cells and NK cells and induces the production of IFN-γ. IFN-γ, in turn, may kill tumor cells directly, inhibit angiogenesis (57–60), and stimulate NK cells, CTLs (61, 62), and macrophages (63) while upregulating MHC I and II molecules (64) on the surfaces of tumor cells.

Fourth, high levels of locally administered IL-12 can reverse tumor-supporting immunosuppression. The immunosuppressive tumor microenvironment is a major hindrance to the clinical efficacy of all cancer immunotherapies. In fact, the cancer vaccine literature teaches that the majority of patients in clinical studies are able to mount significant antigen-specific T cell responses, yet few patients experience clinical benefit (65–67). Similarly, the extraordinary activity of CAR T cells against hematologic malignancies becomes less than ordinary against solid tumors. Many solid tumors lack the chemokines and inflammation necessary to recruit cytotoxic T cells (68). Moreover, dense tumor stroma prevents T cell penetration while immunosuppressive factors released by tumor cells, suppressor T cells and TAMs can cause T cell anergy. Regarding the latter, many tumors bathe in a cocktail of immunosuppressive factors such as TGFβ, IL-10, IDO, and L-arginase. Fortunately, high intratumoral concentrations of IL-12 can cause apoptosis and elimination of CD4^+^CD25^+^Foxp3^+^ suppressor T cells in tumors (69). In addition, the tumor suppressive phenotype of TAMs can be converted to a cytotoxic, antitumor phenotype in the presence of localized IL-12 (11). Finally, IL-12 has been shown to modulate and alter the suppressive activities of tumor-associated MDSCs (12).

Lastly, and perhaps most importantly, activation of T cells in the presence of IL-12 can not only enhance CTL function, but also reduce negative regulatory mechanisms such as PD-1/PD-L1 signaling and autocrine IFNγ-induced apoptosis. This “protective” effect has been observed mostly in the cellular immunotherapy literature. Standard protocols for *ex vivo* expansion of tumor infiltrating lymphocytes for adoptive cell therapy (ACT) traditionally used high dose IL-2 to facilitate T cell proliferation (70). The inclusion of IL-12 in conditioning/expansion media has been explored recently because it had been shown previously to result in optimal T cell priming (71). Indeed, adoptive transfer of tumor-specific CD8^+^ T cells primed *ex vivo* in the presence of IL-12 resulted in enhanced antitumor responses (72, 73), increased persistence of infused T cells (73, 74), as well as increased expression of IL-2Rα (CD25], ICOS, OX40, granzyme B, and IFNγ (73). Importantly, cytotoxic T lymphocytes (CTLs) stimulated with IL-12 were more effective in controlling tumors following adoptive transfer than CTLs stimulated with IFNα (75). IL-12-stimulated T cells expressed lower levels of PD-1 and higher levels of IFNγ and IL-2 compared to IFNα-stimulated T cells (75). IL-12 conditioning caused downregulation of IFNγR2 with a concomitant decrease in susceptibility to IFNγ-induced apoptosis of tumor-infiltrating CD8^+^ T cells (74, 76).

## IL-12 Delivery Strategies

IL-12 delivery strategies can be divided into three general approaches. The first involves fusion of a targeting moiety to IL-12 in order to facilitate accumulation in a tumor following a systemic injection. The most common of class of fusion molecules are immunocytokines, which involve linking a tumor binding antibody fragment to a cytokine. The second approach involves delivery of genetic material encoding IL-12 directly to the tumor or a tissue of interest. This category can be further divided based on the type or method of gene delivery. Plasmids, mRNA, viruses, and transduced cells are all capable of expressing and delivering IL-12 after a local injection. The third major approach involves controlled release of recombinant IL-12 protein from a sustained delivery system. Here, the cytokine delivery system is injected or implanted directly in a tumor or tissue of interest. The remainder of this section will present and discuss the most relevant preclinical and clinical data pertaining to each IL-12 delivery strategy. A summary of current clinical trials utilizing localized IL-12 delivery is presented in Table 2.

**Table 2:** Summary of current clinical trials using localized IL-12 delivery strategies (77).

**Delivery strategy**	**Description**	**Indications**	**Clinical trial identifier**	**Phase**
**Immunocytokines**				
NHS-IL12	Fusion protein of IL-12 and antibody	Advanced solid tumors	NCT01417546	I
		Advanced solid tumors (*with avelumab*)	NCT02994953	I
**Plasmid-based IL-12 delivery**				
GEN-1	Lipopolymer containing pIL-12	Ovarian cancer (*with carboplatin and paclitaxel*)	NCT03393884	I/II
		Ovarian cancer	NCT02480374	I
**Virus-based IL-12 delivery**				
Ad5-yCD/mutTKSR39rep-hIL12	Oncolytic adenovirus-mediated cytotoxic and IL-12 gene therapy	Metastatic pancreatic cancer	NCT03281382	I
		Prostate cancer	NCT02555397	I
Ad-RTS-hIL-12	Inducible adenoviral vector engineered to express hIL-12	Pediatric brain tumor, diffuse intrinsic pontine glioma (*with veledimex*)	NCT03330197	I
		Recurrent/progressive glioblastoma (*with veledimex*)	NCT03679754	I
		R/P glioblastoma (*with veledimex and cemiplimab*)	NCT04006119	II
		Glioblastoma (*with veledimex and nivolumab*)	NCT03636477	I
		GBM, anaplastic oligoastrocytoma (*with veledimex*)	NCT02026271	I
M032	HSV-1 expressing IL-12	Recurrent/progressive glioma, anaplastic astrocytoma, or gliosarcoma	NCT02062827	I
**Cell-based IL-12 delivery**				
CAR-T therapy	Fourth generation CAR-T cells expressing IL-12 (and/or IL-7 and CCL19)	Nectin4-positive advanced malignant solid tumor	NCT03932565	I
**mRNA-based IL-12 delivery**				
SAR441000	mRNA encoding scIL-12, IL-15sushi, IFNα, and GM-CSF	Advanced solid tumors (± *cemiplimab*)	NCT03871348	I
MEDI1191	Lipid nanoparticles (LNP) encapsulating mRNA encoding IL-12	Advanced solid tumors (*with durvalumab*)	NCT03946800	I

### Immunocytokines

As mentioned above, immunocytokines are part or whole cytokines that have been engineered to contain antibody fragments or other targeting moieties. These “targeted” cytokines are administered systemically but are expected to accumulate within tumors at higher levels compared to non-targeted cytokines. Various tumor-related features have been targeted by immunocytokines including: (1) tumor antigens which are overexpressed or uniquely expressed by tumor cells; (2) cryptic extracellular matrix epitopes found only in tumors; and (3) neovasculature markers as tumors require angiogenesis for growth. Developments in immunocytokines are discussed below.

#### Targeting Tumor Antigens

The pan-carcinoma antigen, epithelial cell adhesion molecule (EpCAM), is highly expressed by cancer cells of epithelial origin such as colon, prostate, breast, and lung carcinomas. HuKS-IL-12 is an immunocytokine of IL-12 fused to the Fc fragment of a humanized antibody that recognizes EpCAM. In a murine prostate cancer model, HuKS-IL-12 was found to suppress experimental metastases in SCID mice reconstituted with activated human T and NK cells lymphocytes (78). Another immunocytokine, Hu14.18-IL-12, which is irrelevant in this system due to its targeting of ganglioside GD2, was found to be somewhat less effective than HuKS-IL-12, although differences in antitumor activity were not statistically significant (78). Dual immunocytokines in which both IL-2 and IL-12 were fused to the huKS1/4 antibody fragment were found to eliminate EpCAM-expressing LLC flank tumors following intratumoral (i.t.) injection (79). Interestingly, a mixture of HuKS-IL-2 and HuKS-IL-12 was less effective than the dual immunocytokine if delivered i.t., but exhibited similar in antitumor activity if administered i.v. (79).

The epidermal growth factor receptor HER2/neu is overexpressed in roughly a third of breast and ovarian cancers, with high expression correlating with poor prognosis. Trastuzumab, a monoclonal antibody targeting HER2, has been approved for the treatment of certain breast cancers for more than 20 years. A mouse single chain IL-12 fused to an anti-HER2/neu IgG3 (mscIL-12.her2.IgG3) retarded the growth of CT26-HER2/neu tumors in immunocompetent mice (80). A direct comparison demonstrated that mscIL-12.her2.IgG3 and free IL-12 induced similar activities against CT26-HER2/neu tumors (81). Follow up studies revealed that mscIL-12.her2.IgG3 also displayed robust antitumor activity against MC38/HER2/neu and D2F2/E2 tumors (82, 83). A more recent study revealed that disruption of the heparin binding domain in the mscIL-12.her2.IgG3 immunocytokine, reduced IL-12 bioactivity (84). This result was consistent with recent studies showing that heparin and heparan sulfate bind to and enhance the activity of IL-12 (85–89). While eliminating heparin binding reduces IL-12 activity, we speculate that this reduction could be counterbalanced by an enhancement in tumor targeting as the IL-12 immunocytokine may no longer bind to ubiquitous sulfated glycosaminoglycans in non-targeted tissues.

Mesothelin is a differentiation antigen that is highly expressed in a number of human cancers including mesotheliomas, pancreatic and lung adenocarcinomas, and ovarian and breast carcinomas. To direct IL-12 to mesothelin expressing cancer cells, a scFv, called SS1, that specifically binds to mesothelin was fused to the p35 subunit of a single-chain IL-12 (90). Human peritoneal mesotheliomas established in nude mice were significantly inhibited by i.p. injections of IL12-SS1 (90). That these studies were successful in nude mice seems to imply a prominent role for NK cells in this model.

CA166-9 is a cancer antigen that is expressed in about half of human ovarian cancers (91). A scFv of the 6B11 monoclonal antibody that binds to CA166-9 was fused to mIL-12 (92). Systemically (i.v.) administered 6B11scFv-mIL-12 was found to inhibit the growth of subcutaneously implanted ID8 ovarian tumors more effectively than non-targeted mIL-12 (92).

CD30 is expressed by activated lymphocytes and thus serves as a useful target for several types of lymphoma. A CD30-targeted IL-12 fusion protein was developed for CD30^+^ Hodgkin's lymphoma therapy (93). The immunocytokine was found to induce activation of T and NK cells and secretion of pro-inflammatory cytokines resulting in enhanced cytotoxicity of CD30^+^ MC38 cells. Interestingly, a CD30-targeted IL12-IL2 fusion protein outperformed targeted IL-2 or IL-12 alone in all *in vitro* measures. The dual cytokine construct induced regression of CD30^+^ but not CD30- MC38 tumors *in vivo* (93). Whether the dual cytokine fusion protein was better than the single cytokine constructs is not known as the latter were not evaluated *in vivo*.

#### Targeting Extracellular Matrix

Many solid tumors overexpress extracellular matrix (ECM) which serves as transport barrier to the penetration of therapeutics and immune cells. In ECM-rich solid tumors, it may be impossible to target cancer cells hiding behind layers of ECM. Targeting ECM proteins instead of cancer cells, therefore, is a promising strategy to encourage immunocytokine accumulation in tumors.

There are two immunocytokines, huBC1-IL12 and IL-12-L19, that have been developed to target the splice variant extra domain B (ED-B) of fibronectin, which is highly expressed in tumor tissues but undetectable in normal adult tissues with the exception of endometrium (94). BC-1 is a monoclonal antibody that recognizes the ED-B isoform, thus a huBC1-IL12 immunocytokine has been constructed from two molecules of IL-12 fused to each of the IgG heavy chains of humanized BC-1. Systemic administration of huBC1-IL12 was found to eliminate experimental PC3 metastases and suppress the growth of multiple human tumor lines in immunocompromised mice more effectively than IL-12 alone (95). A Phase I trial evaluated the safety of weekly infusions of AS1409 (huBC1-IL12) in 13 renal carcinoma and malignant melanoma patients (96). The maximum tolerated dose (MTD) was found to be 15 μg/kg. In contrast, the MTD of twice weekly i.v. IL-12 was previously found to be 0.5 μg/kg (97). Dose limiting toxicities, including fever, fatigue, and elevated transaminase levels, were consistent with known toxicities of IL-12 (96).

The second ED-B targeted immunocytokine, IL-12-L19, is comprised of the ED-B-binding L19 scFv and IL-12 (98). L19-targeted cytokines have been shown to selectively accumulate in tumors following i.v. administration (99, 100). Intravenous administration of IL-12-L19 every 48 h was found to control the growth of primary C51 colon adenocarcinomas, F9 teratocarcinomas as well as experimental pulmonary C51 metastasis (101). Biodistribution studies confirmed that a greater percentage of the injected dose of IL-12-L19 was found in tumors as compared to an IL-12-fusion negative control. IL-12-L19 also demonstrated synergistic antitumor activity when combined with L19-TNFα (102).

Most recently, IL-12 was fused to the collagen-binding proteoglycan lumican and mouse serum albumin (MSA), to create IL12-MSA-Lumican (103). Lumican binds to collagen types I and IV, components of the thick fibrotic capsule surrounding tumors and perivascular basement membrane, respectively. In mice bearing established subcutaneous flank B16F10 tumors, treatment with IL12-MSA-Lumican resulted in prolonged tumor control and longer survival. Significant weight loss was observed following IL12-MSA compared to IL12-MSA-Lumican treatment, indicating that collagen targeting may reduce systemic toxicities of IL-12. Finally, the combined treatment of Lumican-MSA-IL2 and IL12-MSA-Lumican potentiated anti-PD-1 increasing survival in multiple models and completely protecting cured mice from live tumor rechallenge (103).

Another collagen-binding immunocytokine comprised of the A3 CBD of von Willebrand Factor fused to both subunits of IL-12 was also recently developed (104). Systemic (i.v.) administration of this CBD-IL12 was found to accumulate in EMT6 mammary carcinomas at significantly higher levels and induce higher rates of complete tumor regression against 1-week old B16F10 and EMT6 tumors compared to IL-12 (104). Inclusion of the CBD resulted in a 5–6-fold decrease in plasma half-life despite the larger size of CBD-IL12. The distributions of CBD-IL12 and IL-12 in normal tissues following i.v. injection were surprisingly similar although typical sites of collagen targeted drugs, e.g., bone and skin, were not examined. Most importantly, although elevated liver enzymes were observed, levels following CBD-IL12 at an impressive dose of 50μg/mouse were similar to 10μg/mouse of IL-12 (104).

In general, the key advantage of systemically administered immunocytokines is their ability to preferentially accumulate within a site of disease, e.g., a tumor. However, immunocytokines retain complete cytokine activity in circulation which allows them to interact with circulating lymphocytes and induce similar cytokine-induced toxicities as parental cytokines (105). One clever strategy has been developed to reduce adverse effects associated with systemic IL-12 by separating the targeted delivery of the p35 and p40 subunits. This split-immunocytokine approach involves first delivering a bivalent p35-based antibody fusion protein (F8-p35S-F8). F8 binds to the alternatively spliced extra domain A (ED-A) domain which is present on the subendothelial extracellular matrix of tumor neovasculature (106). After allowing time for binding and clearance of unbound F8-p35S-F8, a subsequent administration of p40, which has no activity by itself, interacts with p35 to recover IL-12 activity. Quantitative biodistribution investigation in F9 teratocarcinomas bearing mice showed that both targeted subunits accumulated in the tumor (106). Furthermore, the recombined subunits displayed robust IL-12 activity in terms of IFNγ production and STAT4 phosphorylation (106).

#### Targeting Tumor Necrosis

Tumor necrosis is a common feature of most advanced solid tumors. Approaches to target DNA strands that become uniquely exposed in necrotic foci are under investigation. The monoclonal antibody, chTNT-3, recognizes single-stranded DNA (107). A fusion between the variable heavy chain of chTNT-3 and hIL-12 forms the necrosis-targeting immunocytokine, chTNT-3/hIL-12 (94). chTNT-3/hIL-12 was retained in a subcutaneous tumor after i.v. injection and resulted in a significant inhibition of DU145 prostate tumors in human PBL-engrafted SCID mice (94).

Another necrosis-targeting IL-12, capitalizes on the specificity of the NHS76 antibody for ssDNA and dsDNA (108, 109). NHS-IL12 is comprised of the full length NHS76 antibody fused to 2 single-chain IL-12 molecules. Systemic administration of a murine analog, NHS-muIL12, has been shown to delay the growth of MC38-CEA+ colorectal carcinomas in CEA.Tg mice (108). Furthermore, tumor-bearing mice treated with NHS-muIL12 developed CD8^+^ T cell responses against an endogenous tumor antigen, p15E. *In vivo* imaging studies have shown that NHS-muIL12 accumulated in flank tumors following a s.c. injection (108). Subcutaneous administrations of NHS-muIL12 were also recently shown to provide significant reductions in orthotopic MB49luc bladder tumors (110). Tumor control was associated with a noticeable reduction in markers of immunosuppression, e.g., MDSCs, macrophages and tumor-associated TGF-β (110).

The combination of NHS-muIL12 with avelumab, an anti-PD-L1 antibody, resulted in improved control of both MC38 and MB49 flank tumors with higher frequencies of CD8^+^ T cells and enhanced T cell activation compared to either agent alone (111). Against orthotopic EMT-6 mammary tumors (~100 mm^3^) the combination of NHS-muIL12 and avelumab induced complete regression in 7 of 8 mice (112). The same treatment was shown to delay, but not completely regress, the growth of 350–400 mm^3^ established EMT-6 tumors. Importantly, NHS-muIL12 plus avelumab was shown to induce protective immunity as all cured mice resist an EMT-6 tumor challenge but not a 4T1 challenge. Furthermore, treatments enhanced cytotoxic NK and CD8^+^ T-cell proliferation, T-bet expression, plasma cytokine levels, and innate and adaptive immune genes (112).

Combining NHS-IL12 with FcIL-7 or IL-2MAB602 resulted in improved antitumor immunity, increased survival, and long-term remission in sarcoma-bearing mice (113). FcIL-7 is a fusion of interleukin-7 and an Fc fragment while IL-2MAB602 is a fusion of IL-2 and a monoclonal antibody against IL-2, MAb602. Separately, the combination of NHS-IL12 with local tumor irradiation was shown to increase treatment efficacy (114, 115).

In preparation for first-in-human clinical trials, a comparative oncology study in client-owned dogs with melanoma revealed that s.c. injections of NHS-IL-12 induced transient increases in serum IFNγ and IL-10. Two of 7 dogs in a dose escalation cohort experienced a partial response while 5 of 7 dogs had increased levels of tumor-infiltrating CD8^+^ T cells (116).

NHS-IL12 is currently in Phase I clinical studies either as a monotherapy (NCT01417546) or in combination with avelumab (NCT02994953). In the former study, NHS-IL12 induced transient lymphopenia and elevated liver transaminases, but was otherwise well-tolerated with a MTD of 16.8 μg/kg (117). No objective tumor responses were observed, however, 5 of 59 patients experienced stable disease. Immune assays revealed that NHS-IL12 treatment increased NK cell frequencies and broadened the TCR diversity of tumor-infiltrating T cells (117).

#### Limitations

Systemically administered immunocytokines can significantly reduce but are unlikely to completely avoid IL-12-related toxicities. As mentioned above, in circulation, immunocytokines will interact with immune cells and induce signaling outside of the tumor. In addition, all targeting moieties are susceptible to non-specific binding and distribution in normal, untargeted tissues. For example, radiolabeled NHS76 has been found in all major tissues in mice for 2–3 days after i.v. administration (109). Furthermore, substantial amounts of IL-12-L19 were found in the livers of treated animals, likely leading to hepatotoxicity (101).

On-target/off-tissue specific binding may create additional concerns. In the case of NHS-targeting moieties, cancer patients have high levels of circulating cell-free DNA that is shed from tumors (108). It is not clear how circulating DNA impacts NHS targeting. In the case of neovasculature targeting moieties, angiogenesis is a normal process of wound healing and promotes collateral circulation for atherosclerotic blood vessels. Disrupting non-cancerous angiogenesis could induce hypertension and cardiac ischemia which are among the adverse events associated with anti-angiogenic agents, such as bevacizumab.

Moreover, the potential immunogenicity of a non-endogenous immunocytokine is another factor that may limit therapeutic potential. As non-native proteins, immunocytokines could contain immunogenic epitopes against which an immune response, likely an antibody response, could be raised. Anti-immunocytokine antibodies could induce pharmacological abrogation, therapeutic alteration, or hypersensitivity reactions (118). Because of the potential for anti-immunocytokine antibodies, novel immunocytokines should be engineered to minimize the presence of immunogenic epitopes.

Overall, although immunocytokines remain capable of inducing IL-12-related adverse events, the use of targeting moieties may improve biodistribution enough to expand the therapeutic window of IL-12-based immunotherapies.

### Nucleic Acid-Based Delivery

Intratumoral (i.t.) injections of DNA and RNA encoding IL-12 have the potential to localize and sustain the production of IL-12 in the tumor microenvironment. Nucleic acids are much easier to produce, purify and manipulate than recombinant cytokines. However, mammalian host cells are not easy to transfect and typically require chemical, physical, or electrical assistance to achieve reasonable transfection rates. This section will highlight progress in nucleic acid-based approaches both preclinically and clinically.

#### Naked Plasmid

Around the same time that recombinant IL-12 was failing in clinical trials, a limited number of preclinical and clinical studies explored i.t. injection of plasmid DNA encoding IL-12 (pIL-12) as a potentially less toxic approach. Preclinically, i.t. pIL-12 inhibited but did not eliminate B16 melanomas (119). In this study, IL-12 was not detected in the serum following i.t. injection. In another study involving gray horses with metastatic melanoma, i.t. pIL-12 resulted in detectable levels of pIL-12 in the serum for up to 36 h (120). However, it is not clear if systemic dissemination of pIL-12 resulted in significant systemic increases in serum IL-12 or IFNγ as these were not measured (120).

In a Phase I/II trial of intralesional injections with pIL-12, 3 of 9 and 8 of 9 patients with stage IV malignant melanoma experienced clinical and local responses, respectively (121). In a Phase I/IB study, i.t. pIL-12 was found to reduce the size of treated lesions by at least 30% in 5 of 12 malignant melanomas and renal cell carcinomas (122). pIL-12 injections were well-tolerated as no patient in either study experienced a significant treatment-related adverse event. Despite successful safety studies, the use of naked pIL-12 for cancer immunotherapy has not progressed, mostly likely due to poor transfection efficiency.

#### Electric Field-Mediated Gene Delivery

The application of pulsed, high electric fields to facilitate cellular uptake and expression of genes has been a part of the molecular biologist's toolbox for decades. Intratumoral injection of pIL-12 immediately followed by electroporation, referred to here as pIL-12+EP, has been explored in several murine tumor models (123–130). As expected, the benefit of adding electroporation was immediately apparent as one early study showed pIL-12 alone had no effect on B16F10 tumor growth while pIL-12+EP significantly inhibited tumors and extended survival (123). Importantly, the increase in antitumor efficacy was not associated with an increase in systemic IL-12 levels (123).

Among the more notable responses in other early preclinical studies, nearly half of mice bearing established B16F10 melanomas experienced complete tumor regression following 2 weekly treatments with pIL-12+EP (124). In a follow up study, pIL-12+EP induced tumor regression in up to 80% of mice, whereas i.t. injections of pIL-12 alone delayed but could not eliminate B16F10 primary tumors (125). Cured mice displayed protective immunity as 20 of 21 rejected a B16F10 challenge (125).

In the SCCVII squamous cell carcinoma (SCC) model, complete regressions were observed in 40% of mice following pIL-12+EP (127). Furthermore, 3 of 6 cured mice resisted a tumor challenge containing five times the original dose of tumor cells (127, 128). Against BJMC3879 murine mammary adenocarcinomas, CT26 murine colon adenocarcinomas and RENCA renal cell carcinomas, pIL-12+EP significantly suppressed, but did not eliminate implanted tumors (129, 131). Against murine SA-1 fibrosarcomas, pIL-12+EP suppressed tumor growth and induced complete regression in 90% of treated mice with 11 of 18 becoming resistant to tumor rechallenge (132). In this study, IL-12 and IFNγ were detected in the serum of treated mice, however, no side effects were observed (132).

Abscopal responses have been documented in several studies. Against bilateral SA-1 tumors, pIL-12+EP treatment consistently eliminated primary, treated tumors, while slowing the growth of secondary, untreated tumors (132). Similarly, pIL-12+EP treatment of MH134 hepatocellular carcinomas, inhibited both treated and untreated tumors while preventing spontaneous pulmonary metastases (133). A recent study using bilateral B16 tumors demonstrated that an optimized pIL-12+EP protocol (134) was capable of regressing treated lesions while inhibiting the growth of contralateral untreated tumors (54).

Injecting pIL-12 directly into tumors is important as multiple studies have confirmed that i.t. pIL-12+EP treatments were significantly more effective than either peritumoral or intramuscular (i.m.) routes (124, 125, 128, 132). The i.m. route also resulted in significantly more IL-12 and IFN-γ in the serum (124, 128). In terms of mechanism, multiple studies agreed that pIL-12+EP treatment was associated with increased T cell infiltration, increased IFNγ expression and decreased angiogenesis (124, 127, 128, 131, 133, 135). These findings are consistent with known antitumor mechanisms of IL-12.

More recent mechanistic studies have focused on changes in immune cell phenotype and function. For example, B16F10 tumor regression following pIL-12+EP was mediated via the perforin/granzyme lytic pathway while antigen-specific CD8^+^ T cell responses were directed against tyrosinase-related protein epitope Trp2_180-188_ (136). In another study, pIL-12+EP-induced elimination of B16F10 tumors was associated with increased tumor infiltration and polarization of macrophages toward an M1 phenotype (137). Another group found that pIL-12+EP-induced antitumor responses against B16F10 tumors were correlated with a reduction in PD-1 expression on CD4^+^ and CD8^+^ T cells (138). Yet, another group found that the treatment of bilateral B16F10 tumors induced a unique population of CD8^+^ effector T cells with low PD-1 expression in both untreated tumors and systemically (54). This finding suggested that a subset of CD8^+^ effectors generated by pIL-12+EP may be protected or “armored” against checkpoint-mediated exhaustion (54).

A unique feature of pIL-12+EP immunotherapy is the potential to manipulate electric field parameters to enhance transfection, and therefore, efficacy. An exploration of electric field parameters demonstrated that pIL-12+EP-induced cures ranged from 65 to 80% in B16F10 tumor-bearing mice depending on pulsing conditions (138). About half of these cured mice resisted a B16F10 rechallenge (138).

By further enhancing electric field intensity and/or pulse length, it is possible to directly kill tumor cells and release tumor antigens via irreversible electroporation. In one recent study, partial-irreversible electropermeabilization (pIRE) administered after peritumoral electrotransfection with pIL-12 caused complete regression of about 40–50% of treated B16F10 tumors (139). Two of 4 cured mice completely resisted tumor rechallenge while the remaining two experienced delayed tumor growth from the rechallenge. This pIL-12 plus pIRE approach was found to delay, but not eliminate distant, untreated tumors in about half of the mice (139).

There have been several attempts to enhance antitumor activity through the incorporation of additional cytokine-encoding plasmids. Of note, two studies have demonstrated that EP using a combination of IL-12 and IL-18 plasmids outperformed IL-12 alone in terms of antitumor activity (140, 141). The rationale to combine these two cytokines is well-supported given that IL-12 and IL-18 synergize to enhance Th1 responses and IFN-γ production. However, adverse events are also enhanced as systemic co-administration of recombinant IL-12 and IL-18 proteins leads to lethal toxicity in mice (142). In one study, addition of pIL-18 to pIL-12 increased serum IL-12 and IFN-γ levels for at least 6 days after EP although no inflammation was observed in liver, lung, and intestine samples (140). In a second study, pIL-18 did not increase IL-12-induced serum IFN-γ, but intratumoral IFN-γ was significantly higher (141).

Several notable canine clinical studies have explored pIL-12+EP in dogs with naturally occurring tumors. In one such study, pIL-12+EP resulted in a 13–83% reduction in mast cell tumor volume (143). Treated nodules displayed increases in leukocytic inflammation and decreases in the number of malignant mast cells (143). In beagles with canine transmissible venereal tumors (CTVTs), pIL-12+EP induced complete regression of all treated lesions (144). Contralateral untreated tumors were also significantly inhibited. Serum IL-12 levels peaked 7 days after treatment; however, relevant blood chemistries, i.e., liver and kidney enzymes, as well as cell counts were not different from those of control dogs (144). Another study investigated pIL-12+EP for the treatment of canine oral malignant melanoma (OMM). There were no differences in the percentages of helper CD4^+^ and CD8^+^ cells before and after treatment, while T_reg_ frequencies declined from 1.2 to 0.3%. One month post treatment, the objective response rate was 67% (6/9) but by the end of the observation period, all but one of the dogs developed progressive disease (145). A more recent study in 9 dogs with a range of spontaneous cancers, demonstrated that pIL-12+EP induced immunostimulatory and anti-angiogenic effects (146). Administration of three pIL-12+EP treatments every other day caused significant systemic toxicities, including anemia and thrombocytopenia. After switching to a weekly schedule, treatments were well-tolerated, however, all treated tumors continued to progress (146).

Several human clinical trials, mainly against advanced melanoma, have investigated the safety and efficacy of pIL-12+EP. In a Phase I study, 24 patients with stage III or IV melanoma received i.t. pIL-12+EP (six 100 μs, 1,300V/cm pulses) on days 1, 5, and 8 during a single 39-day cycle. Fifty three percent of patients experienced a systemic response, defined as either stable disease or regression of untreated lesions, following pIL-12+EP (147). Most notably, 2 of 19 patients showed complete regression of all metastases. No grade 3 or higher adverse events were observed and neither IL-12 nor IFNγ was detectable in serum samples. In a follow up Phase II study, 29 patients with in-transit or M1a melanoma were treated with up to four 12-week cycles of pIL-12+EP as described above (148). Intermediate results revealed an objective response rate of 33% with 11% complete responses. The treatment was found to increase NK cell levels both intratumorally and systemically (148). No grade 3/4 drug-related adverse events were noted. A subsequent analysis of clinical samples revealed that responses were associated with increased intratumoral infiltration of CD3^+^ T cells (148). In addition, T cell receptor beta chain (TCRβ) sequencing revealed a focusing of the TCR repertoire following treatment. However, there were no differences in T cell clonality between responders and non-responders to pIL-12+EP (148). A second study from the same trial, NCT01502293, found a complete response rate of up to 17.9% and a best overall response rate of 35.7% in patients with stage III/IV melanoma (149). Nearly half of these patients experienced regression of at least one anenestic lesion. An analysis of transcripts in melanoma biopsies found increases in T cell trafficking, immune activation, and antigen presentation (149). Genes associated with adaptive resistance, e.g., PD-L1, TGFβ, and TRAIL, were also increased (149).

Recent results from a safety study in 3 patients with locoregional Merkel Cell Carcinoma (MCC) and 12 patients with metastatic MCC received 1 or up to 4 cycles of pIL-12+EP, respectively (150). The overall response rate in the metastatic MCC cohort was 25% (3/12). Of 10 patients with measurable untreated lesions, 3 experienced abscopal regressions. In addition, 2 patients experienced clinical responses lasting 16 and 55+ months, respectively. Two of the locoregional MCC patients, all of whom were treated with definitive surgery after pIL-12+EP, were recurrence-free at 44+ and 75+ months, respectively (150). Serum IL-12 levels were not measured, but treatments were well-tolerated, and no serious adverse events were observed.

#### DNA Complexes

Lipoplexes, polyplexes, and lipopolyplexes are complexes of lipids, polymers, and lipids plus polymers, respectively, with DNA. These complexes are under investigation to enhance the delivery and transfection efficiency of plasmids encoding genes of interest, including pIL-12. Numerous studies have explored a range of different materials to create novel pIL-12 complexes (151–155). Studies demonstrating antitumor efficacy following local or targeted delivery of pIL-12 complexes are highlighted below.

Polyethyleneimine (PEI), a highly cationic polymer that readily complexes with negatively charged DNA, has been widely used to enhance gene delivery. PEI protects DNA from degradation *in vivo*, encourages interaction with negatively charged cell membranes, and enhances release from lysosomes by acting as proton sponge (156). PEI:IL-12 complexes were shown to transfect lung tissue following delivery via nebulization (157). This approach led to production of IL-12 in the lungs which was not detectable in the plasma of treated mice (157, 158). Weekly or twice weekly administration of aerosolized PEI:IL-12 was found to suppress or eliminate experimental pulmonary metastases of SAOS-2 human osteosarcomas in athymic nude mice (157). Recent attempts to enhance uptake and IL-12 production have focused on modification of PEI with tetraiodothyroacetic acid (tetrac) which binds the α_v_β_3_ integrin receptor that is overexpressed in some tumors (159) or diethylene triamine penta-acetic acid (DPTA) which can reduce the surface charge of PEI:IL-12 complexes (160, 161). As of this writing, no *in vivo* data using either modification have been published.

Polyvinylpyrridilone (PVP) is another cationic polymer that readily complexes with DNA. Twice weekly i.t. injections of pIL-12/PVP complexes were found to eliminate 30 and 50% of 8–10 mm^3^ Renca and CT26 tumors, respectively (162). Most mice that were cured of a primary tumor rejected a subsequent re-challenge (162). In a follow-up study, pIL-12/PVP was found to be more effective than pIFNα/PVP in controlling preclinical tumors, while the combination of pIL-12/PVP and pIFNα/PVP synergized to eliminate 100% of Renca and 50% of CT26 tumors (163). In both studies, CD8^+^ T cells but not CD4^+^ T cells were identified as primary effectors.

Complexation with poly-a-(4-aminobutyl)-L-glycolic acid (PAGA), a biodegradable polyester, enhanced transfection efficiency of pIL-12 and expression of IL-12 *in vitro* and *in vivo* (164, 165). However, T cell infiltration of injected CT26 colon adenocarcinomas and antitumor activities following repeated injections of PAGA/pIL-12 and naked pIL-12 were similar (164).

Encapsulation of pIL-12 in nanoparticles comprised of poly-(D,L-lactic-co-glycolic acid) (PLGA) and 1,2-dioleoyl-3-(trimethylammonium) propane (DOTAP) demonstrated complete regression of up to 75% of established heterotopic BNL hepatocarcinomas following a single i.t. injection (166). Importantly, treatment with encapsulated pIL-12 was more effective than treatment with nanoparticles with pIL-12 adsorbed to the surface (166). Long term expression of inflammatory cytokines could be a concern as both IL-12 and IFNγ were detected in the serum for up to 30 days after treatment (166).

A similar, so-called DMP nanoparticle, comprised of DOTAP and methoxy-poly(ethylene glycol)-poly(lactide) (MPEG-PLA), has been developed to facilitate gene delivery (167). Complexation with pIL-12 resulted in inhibition of CT26 tumors with no signs of systemic toxicity, as determined by appearance, body weight, fecal output, and urinary excretion (167). A slightly different DMP nanoparticle that uses polycaprolactone (PCL) instead of PLA, inhibited the growth of intraperitoneal C26 colon carcinomas and LL/2 Lewis lung carcinomas (168). In addition to delaying tumor growth, DMP/IL-12 particles resulted in high IL-12 gene expression and T cell infiltration although the treatment regimen consisted of 7 daily or every other day treatments (168).

Plasmids complexed with mannosylated chitosan (MC) are under development as a method to target mannose receptors on i.t. DCs. Chitosan is a linear co-polymer of β-linked D-glucosamine and N-acetyl-D-glucosamine. It is primarily derived from the structural polysaccharide, chitin, found in shells of crustaceans. I.t. injection of MC/pIL-12 complexes elicited modest growth delay of CT26 tumors, which was associated with increased tumor cell apoptosis and decreased angiogenesis (169).

Lipopolymers, which incorporate a lipid tail on a polymer backbone, are also under exploration for non-viral gene delivery. Water soluble lipopolymers (WSLP) comprised of cholesterol conjugated to PEI have been developed to increase cell membrane permeability and reduce PEI-mediated toxicity (170). WSLP/p2CMVmIL-12 DNA complexes inhibited the growth of CT26 tumors and improved survival following a single i.t. injection (170). However, the antitumor efficacies of WSLP/pIL-12 complexes and naked pIL-12 appeared similar. In a later study, WSLP/p2CMVmIL-12 complexes injected every 4 days outperformed single injections and multiple injections with naked pIL-12 or PEI/pIL-12 complexes (171). The vast majority of injected WSLP/p2CMVmIL-12 was found in the tumor for up to 24 h with small but increasing accumulation in the liver and blood (171). Subsequent studies demonstrated that i.t. WSLP/p2CMVmIL-12 significantly suppressed the growth of primary and metastatic 4T1, TSA, and EMT-6 mammary carcinomas (172, 173).

Polytraxane (PRX) is a composite molecule made of polyethylene glycol (PEG) and cationic cyclodextrin (CD) that self assembles with DNA into a spherical particle (174). A 4-arm configuration, rather than a linear configuration, had a higher accumulation in MC38-luc tumors and lower accumulation in the lungs following systemic delivery. The 4-arm PRX/pIL-12 also produced higher levels of IL-12 and significantly slowed MC38-luc tumor growth after five i.v. injections of the complex starting 10 days after tumor implantation. (174) Systemic injections of PRX/pIL-12 complexes induced moderate lymphopenia, but no elevation of liver enzymes (174).

pIL-12 complexed with a polyethyleneglycol-polyethylenimine-cholesterol (PPC) lipopolymer was shown to inhibit 4T1 and SCCVII tumors (175) and increase survival in mice with intracranial GL261 gliomas (176) following localized injections. Of note, although the i.p. route is frequently used to deliver drugs systemically, i.p. injections of pmIL-12/PPC for treatment of ID8 ovarian carcinomas resulted in high levels of IL-12 and IFN-γ in ascites but low levels in serum (177). In this study, i.p. pmIL-12/PPC was well-tolerated with no significant changes in serum chemistries (177). In a Phase I study, phIL-12/PPC was administered i.p. to 13 women with chemo-resistant recurrent ovarian cancer (178). Escalating doses of phIL-12/PPC were well-tolerated with no dose-limiting toxicities. Five of the 13 treated patients reported a serious adverse event, however, only one was possibly related to the phIL-12/PPC (178). Similar to preclinical studies, no detectable increase in serum IL-12 was found following phIL-12/PPC treatment (178). A subsequent Phase II study in 20 patients with platinum-resistant recurrent ovarian cancer demonstrated similar safety following weekly i.p. phIL-12/PPC, however, with no objective clinical responses observed (179). A different Phase I trial evaluated the safety of phIL-12/PPC in ovarian cancer patients when combined with carboplatin and docetaxel chemotherapy (180). While there were no dose limiting toxicities, grade 3 adverse events included manageable abdominal pain and cytokine release syndrome. Two of 12 patients experienced complete response while 4 of 12 experienced a partial response (180). A more recent phase I trial combined weekly i.p. pIL-12/PPC with i.v. pegylated liposomal doxorubicin in 14 patients with persistent or recurrent platinum-resistant ovarian or peritoneal cancers (181). Although increased levels of IL-12, IFN-γ, and TNF-α were found in peritoneal fluid, no dose limiting toxicities were observed and a maximum tolerated dose was not reached. The best partial response (28.6%) and stable disease (57.1%) rates were found at the highest dose (36 mg/m^2^) of pIL-12/PPC (181).

pIL-12 complexed with a cationic lipid (+/–)-N-(2-hydroxyethyl)-N,Ndimethyl-2,3-bis(tetradecyloxy)-1-propanaminium bromide/dioleoylphosphatidylethanolamine (DMRIE/DOPE), and injected i.t. was found to inhibit and eliminate CT26 and Renca tumors while protecting up to 96 and 100%, respectively, of mice from rechallenge (182). Interestingly, a direct comparison between naked pIL-12 and DMRIE/DOPE/pIL-12 revealed no difference in antitumor activity (182).

Polyphosphazene particles were modified with hydrophobic N,N-diisopropylethylenediamine (DPA) and hydrophilic monomethoxy poly-(ethylene glycol) (mPEG) to create weakly cationic particles to complex with pIL-12 (183). mPEG/pIL-12 polymersomes delayed CT26 tumor growth when administered i.v. Body weights were unaffected by mPEG/pmIL-12 treatments. Tumor IL-12 levels steadily increased, reaching about 80 pg/g tumor on day 16, while serum IL-12 concentration remained on average about 40 pg/ml after day 9 and continued to day 16. The concentration of IFN-γ in the tumor reached a maximum of 350 pg/g tumor on day 9, while serum levels of IFN-γ slightly increased from day 9 to day 16, reaching a concentration of only 4 pg/ml. While mPEG/pmIL-12 polymersomes did not affect CD3^+^CD4^+^ cells in the tumor, there was a 2-fold increase of CD3^+^CD8^+^ cells and significant increases of CD3^−^NK1.1^+^ and CD3^+^NK1.1^+^ cells in the tumor.

In another study, all-*trans*-retinoic acid (ATRA) was incorporated in cationic liposomes and complexed with pIL-12 (184). ATRA was previously found to increase the expression of TNF receptor 1 and mediate apoptosis of lung cancer cells via TNFα (185, 186). I.v. injections of ATRA-cationic liposome/pIL-12 reduced lung nodules and extended survival compared to cationic liposome/pIL-12 treatment in an experimental pulmonary metastasis model using C26 cells expressing luciferase (184). Concentration of IL-12 in the lungs reached a maximum of 12 pg/mg protein at 6 h post injection, while levels of IL-12 in the spleen and liver were significantly lower and nearly eliminated by 24 h. Interestingly, the incorporation of ATRA reduced liver enzymes levels and thus hepatic toxicity suggesting a possible anti-inflammatory role (184).

#### mRNA-Based Delivery

Recently, mRNA delivery platforms have received tremendous attention, most notably as front running vaccines against SARS-CoV-2. mRNA, like DNA, can encode an unlimited number of proteins and polypeptides. Although mRNA-based platforms are less stable than DNA-based platforms, mRNA can be protected from digestion through encapsulation in polymeric or lipid-based micro- or nanoparticles. A key advantage of mRNA is their ability to directly translate encoded proteins in the cytoplasm. In contrast, DNA must first translocate to the nucleus to be transcribed to mRNA before translation in the cytoplasm.

Regarding the use of mRNA to deliver IL-12 locally, a recent study demonstrated that weekly i.v. delivery of lipid nanoparticles (LNP) loaded with mRNA encoding IL-12 reduced tumor burden in a MYC-driven transgenic mouse model of hepatocellular carcinoma (HCC) (187). The tumor inhibition and extended survival were attributed to increased infiltration of CD3^+^CD4^+^CD44^+^ immune cells and not suppression of MYC (188). Similarly, Moderna/AstraZeneca has developed, MEDI1191, an LNP formulation with IL-12 mRNA. MEDI1191 is currently in phase I clinical trials for intratumoral injection of advanced solid tumors in combination with durvalumab (Table 2) (189). In preclinical studies, a single intratumoral injection of mRNA encoding murine IL-12 (mIL-12) increased IFNγ expression and genes associated with a Th1 response in MC38 tumor-bearing mice (190). When combined with anti-PD-L1, enhanced T cell infiltration and expanded tumor-specific T cell subsets were observed (190).

In another phase I clinical trial, Sanofi and BioNTech are testing SAR44100 (BNT131), an mRNA platform encoding a cocktail of IL-12sc, IL-15sushi, IFNα, and GM-CSF for intratumoral injection as a monotherapy and in combination with cemiplimab (191). To our knowledge, no preclinical data with SAR44100 have been disclosed.

#### Limitations

In general, nucleic acid-based IL-12 delivery approaches are limited by variable transfection rates as well as unregulated production of gene products. In other words, it is easy to control the amount of pIL-12 or IL-12 mRNA delivered but it is not easy to control the dose of recombinant IL-12 that each subject receives. Variable transfection rates may be responsible for conflicting reports on whether pIL-12+EP does (131–133, 140, 143, 144) or does not (124, 126, 130) produce significant increases in serum IL-12 and IFN-γ. Fortunately, lethal IL-12-related toxicities have not been reported in the any of the preclinical or clinical studies detailed above. Nevertheless, the potential for severe IL-12-related adverse events caused by continued and/or unregulated production of IL-12 remains. Strategies to enhance safety and efficacy, either by localizing gene-based IL-12 though incorporation of an anchoring or binding domain or by incorporation of an inducible safety switch to turn off IL-12 production, will help improve the therapeutic window of promising nucleic-acid-based IL-12 delivery technologies.

Another potential limitation that must be considered, is any type of adverse reaction against components of the delivery vehicle or against the nucleic acid vector itself. Regarding the former, foreign delivery components have the potential to induce immune responses which could influence IL-12 delivery. Most notably, it has been reported that about 7 in 10 humans have circulating anti-PEG antibodies (192). The high prevalence of anti-PEG antibodies could limit the efficacy of any PEG-based delivery vehicle. Regarding the immune responses against IL-12 vectors, nucleic acids, particularly DNA that is found in the cytoplasm have the potential to stimulate the cGAS-STING pathway, which may induce its own inflammatory response. Thus, studies utilizing nucleic acid-based delivery must take care to decouple the effects of IL-12 from STING activation.

Lastly, although an exceedingly rare event, DNA vectors could become integrated within a cell's genome. Depending on the site, such integration could have deleterious or even transforming effects. Given that only transient IL-12 expression is desirable, strategies capable for preventing integration, like the use of circular instead of linearized plasmids, should be preferred.

### Virus-Based Delivery

Adenoviruses, Herpes simplex viruses, Semliki forest viruses, poxviruses, and other viral vectors have been engineered to express biologically active IL-12. These engineered viruses injected directly into a tumor are able to infect cancer cells and induce expression of IL-12 within the tumor microenvironment. Furthermore, many viruses have the unique ability to selectively lyse cancers cells after infection. Such oncolytic viruses take advantage of defective cell cycle and interferon signaling pathways that are hallmarks of cancer cells but not normal cells (193). Oncolytic viruses can kill compromised cancer cells in a variety of ways from direct virus-mediated cytotoxicity to indirect destruction of tumor-feeding blood vessels (194). The following sections discuss the progress and limitations of IL-12-encoding viral vectors.

#### Adenoviral Vectors

Adenoviruses are the most well-studied among the IL-12 expressing vectors (47, 195–201). In preclinical studies, i.t. injections of adenoviruses encoding IL-12 (Ad-IL-12) have mediated regressions of murine colorectal carcinomas (202–204), breast carcinomas (47, 201, 202), prostate carcinomas (205, 206) gliomas (207, 208), bladder carcinomas (209), fibrosarcomas (202, 210), laryngeal squamous cell carcinoma (211), hepatomas (212) and hepatocellular carcinomas (213, 214) medullary thyroid carcinomas (215), thyroid follicular cancer (216), and Ewing's sarcoma (217).

Among the more robust responses, Ad-IL-12 induced complete regression of subcutaneous Neuro-2a neuroblastomas in nearly half of mice receiving a single i.t. injection (218). Mice becoming tumor-free also rejected a subsequent tumor rechallenge (218). Similar results were found against CT26 colon adenocarcinomas with more than three-fourths of mice completely eliminating their tumors and all cured mice rejecting a tumor rechallenge (203). The antitumor immune response was mediated primarily by CD8^+^ T cells. Impressively, 3 of 7 mice with bilateral tumors experienced complete regression of an untreated tumor (203). Against 6–23 rat medullary thyroid carcinomas, i.t. injection of AdTCPmIL-12 caused complete regression of more than 60% of treated tumors (215). All cured rats rejected a tumor rechallenge while separate experiments showed that treatment of a single tumor resulted in inhibition of a distant untreated tumor (215). While liver infection following i.t. AdTCPmIL-12 injection was documented, no toxicity was observed (215). A follow up study found similar antitumor and abscopal responses against rat thyroid follicular cancer (216).

In the PyMT-derived transplanted mammary carcinoma model, adenoviral vectors encoding IL-12 induced complete regression in 31% and partial regression in 47% of mice (47). Ten of 11 tumor-free mice completely rejected a tumor rechallenge. In contrast with similar studies using Ad vectors expressing IL-2, no obvious toxic side effects due to AdmIL-12 were noted (47). In another difficult model, a single i.t. injection of Ad.5/3.cRGD-mIL12p70 resulted in >60% long term survival of mice with intracranial GL261 gliomas (207). In a murine model of Ewing's sarcoma (TC71), twice weekly i.t. injections of Ad.mIL-12 significantly delayed treated tumors as well as untreated tumors (217). Ad.mIL-12 also induced complete regression in all treated mice bearing heterotopic MB49 bladder carcinomas (209). Although body weights were not affected, serum IFN-γ levels due to i.t. Ad.mIL-12 were maximal from 2 (~3,500 pg/mL) to 5 days (~1,500 pg/mL) post injection (209).

In a useful comparison against other cytokines, one study demonstrated that Ad-IFN-γ had no greater antitumor activity than an empty Ad vector, whereas AdmIL-12 induced complete regressions of P815 mastocytomas in >80% of treated mice (219). Similarly, Ad-GM-CSF inhibited the growth of FRTL-Tc rat thyroid tumors, however, AdIL-12 was found to be much more effective (220). AdIL-12 also generated systemic immunity capable of inhibiting the growth of distant tumors (220).

An adenoviral vector expressing a single chain IL-12 (scIL-12) was developed to enhance bioactivity over the native heterodimeric form (221). Long-term tumor-free survival was observed in up to 90% of rats with established MH-7777A hepatocellular carcinomas following i.t. infections with Ad.scIL-12 (221). All tumor-free mice were protected from tumor rechallenge. However, despite i.t. injections, IL-12 and IFN-γ were detected in the serum of treated mice (221).

An oncolytic adenovirus expressing single-chain IL-12 (Ad-DHscIL12) was more effective than non-replicating (non-oncolytic) adenoviruses expressing IL-12 at controlling liver metastases of pancreatic cancer in hamsters (222). However, IL-12 levels in serum and non-tumor tissues were similar (222).

In clinical studies, i.t. Ad.IL-12 was well-tolerated with no dose-limiting toxicities in a Phase I trial in 21 patients with advanced pancreatic, colorectal, or primary liver malignancies (223). Serum IFNγ levels peaked 1 day after Ad.IL-12 administration and was likely responsible for the 16 grade 3 adverse events observed. One patient had a partial response and 29% of patients experienced stable disease. Four of 10 assessable patients experienced increases in tumor infiltrating CD4^+^ and CD8^+^ cells. Delayed-type hypersensitivity tests with inactivated adenovirus indicated that all patients developed an immune response against the adenovirus (223).

Despite robust antitumor immune responses, interest in Ad-IL-12 waned in the mid-2000s due to the aforementioned lethal toxicities associated with systemic administration of rIL-12 and the inability of Ad-IL-12, even if administered intratumorally, to prevent systemic dissemination of the cytokine. In recent efforts to mitigate systemic IL-12 dissemination and associated toxicities, two strategies have been developed. The first involves engineering IL-12 to prevent its dissemination. This has been accomplished either by anchoring IL-12 to the surface of tumor cells via fusing a transmembrane domain or glycosylphosphatidylinositol (GPI)-anchored signal sequence to the cytokine (224, 225) or by deleting the N-terminal signal peptide and thus preventing IL-12 secretion (226). Using the former technology, i.t. injection of an adenoviral vector encoding membrane-anchored IL-12 (Ad/scIL-12-B7TM) eliminated the majority of primary CT26 tumors and suppressed the growth of untreated contralateral tumors (5 out of 5 mice), in which complete regression occurred in 1 out of 5 mice (227). Importantly, negligible IL-12 was found in the circulation of mice treated with Ad/scIL-12-B7TM.

The second technology deletes the signal peptide from the p35 subunit of IL-12 to prohibit IL-12 secretion (226). Newly designed oncolytic adenoviral vectors with three genes deleted, i.e., triple deletion (TD), and encoding either wild-type IL-12 (Ad-TD-IL-12) or a non-secreting IL-12 (Ad-TD-nsIL-12) were evaluated in Syrian hamster models of pancreatic cancer. Six i.t. injections of either Ad-TD-IL-12 or Ad-TD-nsIL-12 were found to eliminate subcutaneous HPD1NR tumors in all mice (226). Against peritoneally disseminated SHPC6 tumors and orthotopic Hap-T1 pancreatic tumors, Ad-TD-nsIL-12 outperformed Ad-TD-IL-12 in terms of overall survival (226). Most importantly, Ad-TD-nsIL-12 resulted in significantly lower serum IL-12 levels and reduced systemic inflammatory cytokine expression (226).

The second strategy to mitigate systemic IL-12 dissemination involves conditional expression of IL-12. The Rheoswitch Therapeutic System® (RTS) is an ecdysone receptor-based gene regulation platform in which a transcription factor becomes activated only in the presence of a synthetic small molecule ligand (228). An adenoviral vector encoding the RTS switch and mIL-12 (Ad-RTS-mIL-12) and controlled by the oral activator, veledimex (VDX) was recently shown to extend survival in mice bearing intracranial GL261 gliomas (229). Intratumoral Ad-RTS-mIL-12 plus oral VDX was found to induce IL-12 expression in a dose-dependent manner. Local IL-12 expression correlated with increases in tumor-infiltrating lymphocytes. IL-12 and IFNγ were detected in the sera of treated animals, albeit at an order of magnitude lower than levels found in tumors (229).

Several clinical studies utilizing the regulatable Ad-RTS-IL-12 platform are underway (Table 2). Recent results from a Phase I study in 31 patients undergoing resection of recurrent high-grade glioma demonstrated that VDX induced IL-12 expression in a dose-dependent manner (230). Likewise, the frequency and severity of adverse events, including grade 3 cytokine release syndrome, also increased with VDX dose. Demonstrating the advantage of the inducible system, all serious adverse events were reversible with VDX discontinuation. Interestingly, the use of corticosteroids negatively impacted survival (230). At the optimal dose, and in the absence of corticosteroids, the median overall survival of 17.8 months was encouraging (230). While the localized injection of Ad-RTS-hIL-12 in the resected tumor bed induced IL-12 expression in a recurrent tumor microenvironment, systemic dissemination of IL-12 and its resultant toxicities could not be avoided.

#### Herpes Simplex Virus

Herpes simplex viruses (HSVs) are another family of viruses that have been widely explored for localized IL-12 delivery. Wild-type HSV are cytolytic and thus must be significantly attenuated or rendered replication-incompetent to avoid systemic infection. Injection of replication-incompetent HSV-IL-12 into established hepatomas prior to partial hepatectomy inhibited the engraftment of an intraportal tumor cell challenge in preclinical studies (231). Importantly, no changes in serum IL-12 were detected in treated Buffalo rats (231).

In order to capitalize on their lytic potential, HSVs have been engineered, through deletion or mutation of genes responsible for viral replication such that only cancer cells are lysed. Not surprisingly, these oncolytic HSVs (oHSVs) have been shown to exhibit greater antitumor potential than their replication-incompetent, parental counterparts. For instance, treatment of established hepatomas with a non-cytokine encoding oHSV exhibited significant antitumor activity which was further increased with an IL-12 insert (232). The oHSV-IL-12 treatment was also more effective than oHSV at protecting animals from a tumor rechallenge (232).

Similar antitumor responses to oHSV and increased efficacy with oHSV-IL-12 were observed against flank CT26 and SCCVII tumor models (233–235). A single i.t. injection was able to delay the growth of established SCCVII tumors whereas multiple injections induced complete regressions in 5 of 6 treated mice (235). In contrast, multiple injections of oHSV-GM-CSF cured only half of treated mice (235). In the CT26 model, oHSV-IL-12 inhibited or eliminated injected 5 mm tumors while inhibiting non-injected, contralateral tumors (236, 237). When low dose mIL-12 was added to i.t. oHSV-IL-12, both treated and untreated CT26 tumors were eliminated in more than two-thirds of mice (237). The importance of T cells in this model was established as oHSV-IL-12 had no antitumor effect in CT26-bearing nude mice (236, 237).

Intraperitoneal administration of oHSV-IL-12 also increased survival in MISIIR-TAg mice bearing ovarian carcinomas (238). Treatment was associated with tumor antigen-specific CD8^+^ T cell infiltration (238). Against flank SARC-043 and SARC-045 sarcomas, oHSV-IL-12 extended survival compared to saline injections (239). Although there was no difference is survival between control and IL-12 encoding oHSV, the oHSV-IL-12 was found to increase tumor-infiltrating effector T cells while decreasing immunosuppressive MDSC and T_reg_ populations (239).

In an interesting comparison of cytokines, oHSV-IL-12 was more effective at inhibiting flank prostate tumors, TRAMP-c2 and Pr14-2, than oHSV encoding GM-CSF (oHSV-GM-CSF) which was no more effective than non-cytokine encoding oHSV (240). Only 1 of 18 mice treated with oHSV-IL-12 exhibited an increase in serum IL-12 4 days after treatment (240). oHSV-IL-12 also significantly outperformed oHSV-GM-CSF in a model of CT26 metastasis (241). In addition to localizing IL-12, i.t. injections may be key in assuring safety as intrasplenic injections of oHSV-IL-12 induced concerning increases in IL-12 and IFNγ in both serum and liver specimens (241).

Frequent and worthwhile targets of oHSV-IL-12 immunotherapy are the various forms of brain cancer. In one early study, oHSV-IL-12 was found to extend survival and cure approximately one-fourth of mice bearing 5-day-old intracranial Neuro-2a neuroblastomas (242). Treatment was associated with an influx of CD4^+^ and CD8^+^ T cells as well as macrophages. The inclusion of IL-12 was critical, as median survivals of mice treated with the parental, non-cytokine encoding oHSV or saline were similar (242). Likewise, oHSV-IL-12 but not non-cytokine oHSV was effective at extending survival against intracerebral mouse 005 glioblastomas (243). In an intracranial 4C8 glioma model, oHSV-IL-12 was significantly more effective than oHSV at extending survival and curing mice (244). In a model of breast cancer metastasizing to the brain, i.t. oHSV-IL-12 modestly extended the survival of mice bearing intracranial SCK tumors (245). Intracerebral injections of oHSV-IL-12 in owl monkeys resulted in neither histopathological changes in brain tissue nor clinical evidence of toxicity, as assessed by changes in temperature, neurologic performance, feeding or social behavior, or weight (246).

In addition to encoding cytokines, HSVs can be engineered to target cancer-associated antigens. Four i.t. injections of a HER2-targeted oHSV-IL-12 was significantly more effective at inhibiting both day 3 and day 10 tumors than the non-cytokine encoding parental HSV (day 3: 15/22 oHSV-IL-12 vs. 7/20 HSV becoming tumor free; day 10: 3/18 oHSV-IL-12 vs. 1/12 HSV becoming tumor free) (247). Immune responses to oHSV-IL-12 included elevated levels of IFNγ, IL-2, Granzyme B, Tbet, and TNFα as well as Th1 polarization and NK activation (247). This HER-2 targeted oHSV-IL-12 was also found to induce complete remission in more than one-fourth of treated mice bearing orthotopic high grade gliomas (HGG) expressing HER2 (248). Cured mice were protected from HGG rechallenge regardless of HER2 expression (248). This is an important finding given the heterogeneity of HER2 expression among and within tumors.

The clinical precedence for HSV has been established with Talimogene laherparepvec, which encodes for GM-CSF and is approved for intratumoral injection in patients with advanced, non-resectable melanoma. A clinical grade preparation of HSV-1 encoding hIL-12 (M032) induced no adverse clinical signs after intracerebral injection in non-human primates (249). A Phase 1 clinical trial exploring the safety of M032 in patients with recurrent or progressive glioblastoma multiforme, anaplastic astrocytoma, or gliosarcoma is currently recruiting [NCT02062827].

#### Semliki Forest Viruses

Semliki Forest Virus (SFV) is an alphavirus that was first isolated from mosquitos in Uganda and has a broad range of hosts, making it ideal for translation (250). In addition, modified SFV can produce higher levels of recombinant protein than retroviral vectors, and expresses protein more stably than adenoviral vectors (251). SFV is also less pathogenic in humans, and induces apoptosis of tumor cells at the end stage of virulence (250).

SFV encoding IL-12 (SFV-IL-12) was found to extend the survival of mice with established orthotopic 203 gliomas (252). Because SFV infection induces apoptosis, uptake of infected tumor cells by dendritic cells in the presence of IL-12 is posited as a potential mechanism of enhanced antitumor activity. In a B16 brain tumor model, the same group demonstrated a prolonged median survival by 5 days when immunizing with SFV-IL-12 pulsed dendritic cells, compared to a retroviral vector encoding IL-12 (253).

In a woodchuck HCC model, induced by chronic infection with woodchuck hepatitis virus (WHV), using a single intratumoral treatment with SFV-enhIL-12, which included 10 separate injections at different sites of one tumor, partial tumor regressions of up to 80% occurred in 5 out of 6 animals (254). Although all tumors regrew after treatment, injections of SFV-enhIL-12 resulted in a favorable safety profile with only transient reductions in body weight (254).

In a transgenic mouse model of spontaneous HCC, i.t. SFV-IL-12 treatments achieved 100% survival for at least 135 days (255). Interestingly, SFV-IL-12, which induces transient infection and expression of IL-12, was found to be more effective and less toxic than long-term IL-12 expression induced by a plasmid encoding IL-12 which was delivered hydrodynamically (255).

I.t. SFV-IL-12 inhibited tumor growth and extended survival in mice bearing orthotopic 4T1 mammary carcinomas (256). When administered neoadjuvant to resection and combined with attenuated *Salmonella* (LVR01) as a post-surgery adjuvant, an impressive 90% long-term tumor free survival was achieved (256).

While there is consensus on its benefits, the mechanism of SFV-IL-12-induced tumor regression is debated. Initial studies performed in a subcutaneous B16 melanoma model did not find a significant decrease in tumor regression when using T cell-deficient nude or NK cell-deficient beige mice. Rather, this group suggested that inhibition of angiogenesis mediated by IFNγ production causes massive tumor necrosis (257). A decade later, the same group further supported this conclusion using iNOS deficient mice that express high levels of VEGF (258). The effect of IL-12 was even more pronounced, with fewer tumor vessels in iNOS^−/−^ mice than in wild-type mice given the same treatment with SFV-IL-12 (258). However, they did find immunohistochemical evidence that NK cell activation and recruitment is correlated with murine endothelial cell death (258). More recently, the efficacy of SFV-IL-12 was found to be dependent on type I interferons produced by macrophages and dendritic cells (259). Furthermore, the type I interferon receptor, IFNAR, is necessary for IL-12-dependent CD8^+^ T cell expansion (259). Different routes of administration were compared using a subcutaneous P815 model and demonstrated that i.t. injection of SFV-IL-12 was superior in producing IFNγ compared to either s.c. or i.v. routes (260). Importantly, none of the SFV-IL-12 injections resulted in increased serum levels of IFNγ (260).

The combination of SFV-IL-12 with monoclonal antibodies or adjuvants is another strategy that has been studied. By administering SFV-IL-12 and anti-CD137 to provide co-stimulation to T cells, survival was improved in both s.c. B16-OVA and s.c. TC-1 models with a maximum long-term survival of 75% in both models (261). In the bilateral B16-OVA model, 90% of treated and 22% of untreated tumors experienced complete regression with 10^8^ SFV-IL-12 viral particles and anti-CD137 (261). CD8^+^ T cells were crucial for this tumor regression and SFV-IL-12 increased the ratio of CD8 T/T_regs_ compared to anti-CD137 alone. A subsequent paper further demonstrated that SFV-IL-12 induces PD-L1 expression on B16-OVA cells and therefore combined PD-1/PD-L1 blockade with the viral construct (262). This combination significantly enhanced survival compared to individual components using B16-OVA and MC38 models, with long-term survival >75% (262).

Modifications to improve the performance of SFV-IL-12 vectors have also been explored. An enhanced (SFV-enhIL-12) vector with separate promoters for each subunit of IL-12 increased IL-12 expression 8-fold over the single promoter construct (251). One i.t. injection of 10^8^ viral particles using either the original or enhanced constructs resulted in >80% long-term tumor free survival of MC38 tumor-bearing mice (251). Lower doses of SFV-enhIL-12 induced tumor regression more efficiently than SFV-IL-12, although the enhanced vector induced higher levels of serum IL-12 (251).

A separate enhanced SFV vector with 10x higher gene expression, called pSFV10-E-IL12, has also been developed (263). This vector also included separate promoters for p40 and p35 with an additional capsid enhancer to drive enhanced expression of the recombinant protein. Two helper vectors with instructions for structural proteins were cotransfected with the enhanced SFV to produce virus-like particles (VLPs). Intratumoral injections in subcutaneous K-BALB and CT26 tumors caused complete regression and furthermore inhibited primary tumor growth and reduced metastases in a heterotopic 4T1 model (263).

In an effort to limit undesirable virus-mediated cytotoxicity for intracranial applications, SFV VLPs expressing IL-12 have been developed (264). Low dose (5 × 10^7^ VLPs) treatment decreased orthotopic RG2 rat glioma volumes by 70% and extended survival by a little more than 20% over vehicle controls. High dose (5 × 10^8^ VLPs) treatment resulted in enhanced tumor reduction but also caused CNS inflammation, necrosis, and treatment-related death (264). The broad infectivity of SFV-based vectors is a major limitation to their use in CNS applications.

#### Poxviruses

Vaccinia virus (VV), which was used extensively during the eradication of smallpox, has a large capacity for gene insertion, a wide host range and high gene expression efficiency. VV expressing IL-12 (VV-IL-12) has been found to inhibit, but not eliminate, C6 gliomas in nude mice following i.t. injection (265). Low VV-IL-12 doses, 10^2^-10^4^ pfu, and high VV-IL-12 doses, 10^5^-10^7^ pfu, resulted in similar levels of tumor inhibition (265). All doses above 10 pfu resulted in cytokine-associated toxicities punctuated with a 40% mortality rate in the high dose group (265). Similar tumor inhibition and mortality were found in a subsequent publication which also correlated high plasma levels of IFNγ and TNFα with toxicity (266). VV-IL-12 and IL-2 expressing VV (VV-IL-2) exhibited similar inhibition of C6 gliomas (265, 266) However, against AE17 mesotheliomas, VV-IL-12 cured 80% of mice compared to only 20% of mice treated with VV-IL2 (267). Recently, an oncolytic VV-IL-12 was shown to increase lymphocytic infiltration and inhibit LLC and B16F10 tumors with 14–25% complete responses (268). Antitumor efficacy was enhanced by the addition of an IL-7 expressing VV (VV-IL7) as well as immune checkpoint inhibitors (268). The combination of VV-IL-12 and VV-IL7 did not affect mouse body weight (268) and appears to be better tolerated than previous VV-IL-12 treatments which utilized different VV strains (265, 266).

A recombinant canarypox virus (ALVAC) encoding IL-12 has been shown to induce complete regression of TS/A mammary carcinomas after six i.t. injections, whereas a single i.t. injection of ALVAC-IL-12 had no effect on tumor growth (269). Of the mice that were rendered tumor-free, 70% were protected from a contralateral rechallenge. Antitumor activity was driven by IL-12 as the ALVAC vector itself displayed very limited tumor inhibition. In a Phase I study in 9 patients with unresectable metastatic melanoma, i.t. injections of ALVAC-IL-12 resulted in increased intratumoral IL-12 mRNA expression in only 4 of 9 patients (270). Three patients experienced modest increases in serum IL-12 and IFNγ levels and no dose-limiting toxicities were observed. One patient had a complete response of an injected lesion and an uninjected in-transit metastases (270). In a similar Phase I study performed by the same group, only 1 of 4 evaluable patients exhibited an increase in intratumoral IL-12 mRNA after ALVAC-IL-12 administration (271).

#### Other Viruses

Attenuated measles viruses (MeVac) have been used safely as a vaccine to prevent measles in billions of people. MeVac is another type of oncolytic virus which makes it an attractive potential cancer therapy. MeVac encoding IL-12 (MeVac FmIL-12) achieved complete regression of carcinoembryonic antigen (CEA) expressing MC38 tumors in 90% of mice treated i.t. (272). MeV encoding anti-PD-L1 or IgG-Fc induced complete regressions in 60 and 40% of treated mice, respectively. Antitumor activity was CD8^+^ T cell dependent and correlated with high levels of intratumoral IFNγ. All mice experiencing complete regressions following MeVac FmIL-12 also complete rejected tumor rechallenge (272). A follow up study demonstrated that MeVac FmIL-12 was more effective at controlling tumors than MeV encoding IL-15 in two murine tumor models (273). Against MC38-CEA tumors, MeVac FmIL-12 eliminated tumors in all treated mice whereas MeV-IL15 induced complete tumor regressions in 60% of mice (273). Effects appear to be model dependent as treatment of B16 tumors expressing human CD46 with MeV-IL-12 and MeV-IL15 only modestly extended survival (273).

Vesicular stomatitis virus (VSV) is another potent oncolytic virus under exploration for cancer therapy. VSVs engineered to express IL-12 (VSV-IL-12) were recently evaluated in an orthotopic floor of mouth model of SCCVII murine squamous cell carcinoma (274). Multiple i.t. injections of VSV-IL-12 were more effective at eliminating tumors and induced higher long-term survival rates (40 vs. 0%) compared to non-cytokine encoding VSV (274).

Moloney murine leukemia virus (MoMLV) is a positive sense, single-stranded RNA (ssRNA) retrovirus. A MoMLV expressing IL-12 was engineered to display an anti-erbB2 scFv against the HER2 receptor (275). Eight daily i.t. injections of 6-day-old flank MBT2 tumors was found to result in inhibition and complete regression in about one-fifth of mice treated with the HER2-targeted MoMLV-IL-12 (275).

A hybrid viral vector which pairs the high infectivity of adenoviruses with the high gene expression of SFV has been engineered to specifically infect and express IL-12 in hepatocellular carcinoma cells (276). In Buffalo rats bearing orthotopic McA-RH7777, the AdV-SFV hybrid vector expressing IL-12 induced complete regression in 4 of 12 treated animals. While the non-hybrid SFV-IL-12 induced complete regression in 50% of treated rats, treatment was accompanied by elevated liver enzymes indicating hepatotoxicity (276). In contrast, liver enzyme levels following administration with the tissue-specific, hybrid vector were no different than levels in saline-treated control rats (276).

Newcastle disease virus (NDV) is an oncolytic, enveloped, negative-sense, single-stranded RNA virus. NDVs have been engineered to express checkpoint inhibitor molecules and checkpoint inhibitor-cytokine conjugates (277). Although the antitumor activity of i.t. NDV expressing IL-12 alone was not tested, 5 i.t. injections of NDV-anti-CD28-mIL-12 plus i.p. anti-CTLA-4 or NDV-antiPDL1-mIL-12 plus i.p. anti-CTLA-4 resulting in complete responses in 77 or 70%, respectively of mice bearing 9 day-old B16F10 flank tumors (277). The same treatments of one of two bilateral tumors resulted in 10 and 62% complete responses, respectively, of untreated tumors (277).

#### Limitations

A major benefit of virus-based IL-12 delivery is high transfection rates, particularly when compared to non-viral vectors. However, virus-based delivery is limited in several aspects. First, neutralizing immune responses including anti-viral antibody production (271, 278, 279) and DTH responses (223) may preclude repeated injections with the same vector. However, the extent to which anti-viral immunity limits repeated injections is unclear. Mice previously exposed to a viral vector demonstrated a 2.4-fold reduction in intratumoral transgene expression. This level of reduction did not affect antitumor activity; however, the potential exists for reduced antitumor immune responses when lower doses of virus are used (280). On the positive side, viral dissemination and transgene expression in non-targeted liver tissue was reduced by more than 1,000-fold in mice pre-exposed to the viral vector (280).

Secondly, viral IL-12 delivery requires transfection of host cells, which has been shown to be highly heterogeneous due to inherent susceptibility or previous exposure to related vectors (223). In an extreme example of susceptibility, a dose of AdCMVmIL-12 that was found to be lethal in 100% of treated C57BL/6 mice did not result in a single death in the Balb/c strain (281). Livers of C57BL/6 mice exhibited much higher levels of adenovirus transduction (281). The current coronavirus outbreak further illustrates the point that some individuals appear to be more susceptible than others to viral infections.

Third, although clinical trials have shown that recombinant viruses are tolerated, viral dissemination, and infection in liver cells following i.t. injection were reported at an alarming degree (282–284). Even when small volumes (10–20 μl) of recombinant adenoviruses were injected i.t., transgene expression was found in the liver, intestine, spleen, kidney, and brain. As much as 34% of the injected AdV infected non-targeted normal tissues (284). Similar levels of transgene expression was found in the liver and tumor 3 days after an i.t. injection (280).

Fourth, some viral vectors, such as MoMLV and MeV, can insert their genetic information into an infected cell's genome causing undesirable mutations and uncontrolled long-term expression of IL-12. The benefits of using such integrating vectors must be weighed against these risks as other viruses, such as AdV, HSV, VSV, and NDV, are non-integrating and induce transient expression (285).

Lastly, and most relevant to the topic of localizing IL-12, even local injections of viruses encoding IL-12 are not able to prevent IL-12 dissemination (47, 202, 282, 286, 287). Consequently, antitumor responses and IL-12-mediated adverse events are strongly correlated with virus dose. Similar to plasmid-based IL-12 delivery, novel strategies that anchor IL-12 to an injection site or prevent its secretion appear best suited to break this correlation and widen the therapeutic window of virus-encoding IL-12.

### IL-12-Transduced Cells

Dendritic cells (288, 289), fibroblasts (290, 291), macrophages (292, 293), tumor cells (294, 295), and mesenchymal stem cells (MSCs) (296–298) have all been engineered to express IL-12. Transduced DCs, fibroblasts, tumor cells, and macrophages have been directly injected into tumors, while MSCs have been injected systemically for migration to tumor beds (296, 297). A much larger number of preclinical and clinical studies have used IL-12-transduced tumor cells for s.c. immunization. These studies are not covered in this review as IL-12 in this context is functioning as a vaccine adjuvant following injection of transduced cells in healthy, non-cancerous s.c. tissue rather than a strategy to localize IL-12 to a tumor.

#### Dendritic Cells and Macrophages

Dendritic cells and macrophages have been engineered to express a variety of cytokines including IL-12. In addition to supplying the tumor microenvironment with IL-12, transduction of these antigen presenting cells increases their ability to induce robust antitumor immune responses. Following i.t. injection, DCs and macrophages are capable of capturing tumor antigen, migrating to draining lymph nodes, presenting antigen and priming tumor-specific T cell responses. Transfection of antigen presenting cells with IL-12 helps polarize T cells to a Th1 phenotype and facilitate robust tumor-specific CTL responses (288).

In an early preclinical study, bone marrow-derived dendritic cells (BMDCs) expressing IL-12 were more effective than DCs expressing IL-2 in controlling the growth of B16F10 melanomas and generating tumor-specific CTL activity (299). IL-12-transduced BMDCs were also found to significantly suppress established MCA205 fibrosarcomas, B16 melanomas, D122 adenocarcinomas, and 178-2 BMA prostate carcinomas following a single i.t. injection (288, 300, 301). In one study, 36% of treated MCA205 tumors were completely rejected. Tumor-free mice also rejected a subsequent MCA205 rechallenge (288). The growth of contralateral, non-treated tumors was also suppressed, implying that systemic tumor-specific immunity can be induced following i.t. injection. IL-12-transduced BMDCs were substantially more effective than IL-12-transduced fibroblasts or non-transduced BMDCs at inducing tumor-specific CTL activity and IFNγ production by draining lymph node cells and splenocytes (288). Enhanced trafficking of DCs to regional lymph nodes provides a major advantage over non-migratory cells such as IL-12-transduced tumor cells or fibroblasts.

DCs transfected with adenovirus to express IL-12 (DC-AdCMVIL-12) were found to eliminate CT26 and MC38 colon adenocarcinomas in 67–84% and 50% of mice, respectively (289, 302). Mice experiencing complete regression exhibited tumor-specific CTL activity and rejected tumor rechallenge. I.t. injections of AdCMVIL-12-infected fibroblasts or allogeneic DC-AdCMVIL-12 were not effective (289). In a similar study, DC-AdCMVIL-12 reversed immune suppression mediated by TBJ neuroblastomas and induced complete tumor regression (303).

DCs engineered to co-express both IL-12 and IL-18 displayed synergistic antitumor activity that was greater than either DC-encoding cytokine alone (304). All mice experienced complete regression of CMS4 tumors while treated MethA tumors were substantially reduced (304). While serum IFN-γ levels reached 400 pg/ml 2 days after DC administration, no toxicities were noted (304).

BMDCs transfected by simian virus 40 (SV40) to express IL-12 or IL-15 demonstrated complete regression of CT26 colon adenocarcinomas in 40 or 73% of mice, respectively (305). There was no synergy between IL-12 and IL-15 when delivered together. Interestingly, maturation of DCs with LPS prior to i.t. injection impaired antitumor activity, presumably due to reduced antigen loading in the tumor. However, this hypothesis was not evaluated (305).

Transfection of BMDCs isolated from CMS4 sarcoma-bearing mice with an adenoviral vector expressing IL-12 was shown to restore antigen-presenting and allostimulatory functions (306). Direct injections of the rescued BMDCs were shown to eliminate intrahepatic CMS4 tumors and induce protective immunity (306). DCs can also be isolated from mouse spleens prior to transduction with an IL-12 encoding vector. A recent study demonstrated that splenic DCs overexpressing IL-12 can inhibit the growth of melanomas in mice (307).

IL-12-transduced macrophages are less well-studied, however, like DCs, macrophages have antigen presenting capabilities that can be enhanced by IL-12. A single i.t. injection of peritoneal exudate-derived macrophages transduced by AdmIL-12 induced suppression of primary and metastatic 178-2 BMA orthotopic prostate tumors (293). Serum IL-12 levels peaked 3 days after injection and remained elevated for at least 2 weeks. Antitumor activity was correlated with increased leukocytic infiltrate and cytolytic activity (293). The AdmIL-12-transduced macrophages were found to migrate to draining lymph nodes following i.t. injection.

Similar to some viral constructs, to reduce the potential for toxicity, IL-12 expression can be placed under the control of an inducible promoter. One inducible system consists of 2 cassettes including Gal4-EcR;VP16-RXR and IL-12. First, Gal4-EcR and VP16-RXR are expressed under the constitutive ubiquitin C promoter. The heterodimerization of Gal4-EcR and VP16-RXR are triggered by addition of small-molecule ligand, e.g., pharmacologically inert diacylhydrazine. Gal4-EcR-VP16-RXR heterodimers bind to a synthetic promoter containing Gal4 binding site and induce the expression of IL-12 (308). DCs with inducible IL-12 demonstrated inhibition of Renca and MethA tumors following i.t. administration (309). Combining IL-12-expressing DCs with either IL-21- or IFNα expressing DCs did not improve antitumor efficacy (309). The ability to turn cytokine expression “on” and “off” is an attractive feature which may potentially allow for better control over cytokine delivery.

To date, only one clinical study has been published. In a Phase I study, autologous dendritic cells transfected to express IL-12 were administered i.t. to patients with metastatic gastrointestinal carcinomas (310). Of the 17 treated patients, one experienced a partial response and two experienced stable disease. Serum IFNγ, but not IL-12, levels were elevated following treatment. Four instances of transient grade 3 lymphopenia were observed, but no grade 4 toxicities were observed. *In vitro* IL-12 production was highly variable, most likely due to differences in susceptibility of dendritic cell infection with adenoviruses encoding IL-12 (310).

#### Fibroblasts

Fibroblasts expressing IL-12 caused the elimination of 7-day MCA207 tumors in 70–100% of mice after a single peritumoral injection (291). In addition, local IL-12-mediated regression was also able to control tumors on the contralateral flank. As has been shown in other systems, i.t. injections were more effective than distant s.c. injections at controlling tumor growth. Repeated injections of IL-12-engineered fibroblasts were well-tolerated, with no abnormalities detected in liver and renal function tests (291). A similar peritumoral implantation of alginate encapsulated NIH3T3 fibroblasts expressing IL-12 was found to inhibit CT26 tumor growth (311).

In a Phase I study, peritumoral injections of IL-12-transduced autologous fibroblasts induced transient tumor reductions in four of nine patients with disseminated cancers (312). Treatment with fibroblasts capable of secreting up to 5,000 ng of IL-12 per day was well-tolerated (312).

#### Mesenchymal Stem Cells

Mesenchymal stem cells (MSCs) are multipotent adult stem cells that divide and differentiate in order to repair skeletal tissues, such as bone, cartilage, and muscle. As such, MSCs are predisposed to traffic from bone marrow to a wound site based on expression of soluble factors and unique receptors in damaged tissues. Tumors can express many of the same factors and receptors which results in MSCs trafficking to, and infiltrating tumors following systemic injections (313). Thus, using IL-12 transduced MSCs as a “Trojan horse” to target tumors and deliver IL-12 is a promising approach.

In one preclinical study, Ad.IL-12-transduced MSCs inhibited the growth of TC71 human Ewing sarcomas in mice (314). One potential concern about IL-12-expressing MSCs (MSC/IL-12) is their tropism for some normal tissues. In the above study, MSC/IL-12 were found in tumor, liver, lung and spleen tissues but not kidney, heart, or brain tissues 10 days after i.v. injection (314). In a different study, high levels of MSC/IL-12 were found in the tumor but not in the liver, lung, and spleen 20 days after administration (297). Earlier time points were not presented. IL-12 levels were high in the tumor; however, significant serum IL-12 levels were found to persist for at least 2 weeks after MSC/IL-12 administration (297). These high levels of intratumoral IL-12 resulted in the inhibition of 786-0 renal cell carcinomas in nude mice (297).

In a study that illustrates the importance of tropism, MSCs that were non-virally transfected with pIL-12 significantly inhibited B16F10 lung metastases but not subcutaneous B16F10 tumors following i.v. administration (315). Only when administered i.t., were MSC/IL-12 cells able to inhibit s.c. B16F10 tumors (315). Despite the tumor-homing potential of MSCs, published data suggest that MSC/IL-12 may be most effective at treating tumors in tissues where MSCs have a natural tropism, e.g., lung, liver and bone (315).

Several other studies have favored local over systemic administration of MSC/IL-12 to treat various tumors. Intracranial GL26 glioma-bearing mice treated with i.t. UCB-MSC-IL12M generated from human umbilical cord blood experienced significantly prolonged survival (316). Specifically, 70% of UCB-MSC-IL12M treated mice survived more than 90 days post tumor implantation whereas all mice treated with MSCs expressing GFP succumbed within 65 days (316). Cured mice were completely protected from ipsilateral and contralateral tumor rechallenge (316). In another study, bone marrow derived MSCs were transfected with a lentivirus to express mIL-12 (317). These Lenti-mIL-12-MSCs reduced the volume of H22 and MethA ascites and increased survival when administered i.p. 2 and 7 days after tumor inoculation (317). No pathological abnormalities were noted on biopsies taken from internal organs of Lenti-mIL-12-MSC treated mice (317).

Human MSCs transduced with a retroviral vector expressing a modified IL-12 (MSCs/IL-12M) inhibited B16F10 melanomas following five i.t. injections over 3 weeks (298). Safety was implied as no IL-12 was detected in the serum following treatment. Interestingly, the antitumor activities elicited by i.t. injections of MSCs/IL-12M and IL-12 plasmid DNA were similar (298).

In a rare comparison of cytokine delivery technologies, i.t. injections of MSCs transfected with rAd/IL-12M inhibited B16F10 primary and metastatic tumors more effectively than i.t. injections of rAd/IL-12M alone (318). Despite the homing feature of MSCs, i.t. injections displayed greater antitumor activity than i.v. injections of MSCs/IL-12M. Similar to the study by Gao et al., serum levels of IL-12 and IFN-γ were persistently elevated following i.t. administration of MSCs/IL-12M (318).

#### Tumor Cells

TSA mammary carcinoma cells transduced to secrete IL-12 were found to cure 10 of 40 mice bearing 1-day established TSA tumors following local injection (294). When tumors were allowed to establish for seven days prior to treatment, antitumor efficacy dropped, with only 2 of 40 mice experiencing tumor regression.

Local delivery of 9L gliosarcoma cells engineered to express IL-12 (9L-IL12) significantly prolonged the survival of rats challenged intracranially with wild-type 9L tumors (295). Similar results were observed whether the 9L-IL12 treatment was given at the same time or 5 days later than the tumor inoculation. The authors noted that only mice receiving the delayed treatment were protected from tumor rechallenge (295).

#### Other Cells

Natural killer (NK) cells and chimeric antigen receptor (CAR) T cells have also been transduced to produce IL-12. In one recent study, primary NK cells isolated from C57BL/6 mice and transduced to express IL-12 (NK^IL−12^) increased MHC class I expression in Ova-transfected melanoma cells (B16M05) compared to mock plasmid transduced NK cells (319). Combination treatment studies involving tumor-specific cytotoxic T lymphocytes (CTLs), PEGylated IL-2, and activated NK^IL−12^ revealed that only groups containing activated NK^IL−12^ demonstrated statistically significant prolonged survival over mock constructs, untreated, and CTL alone controls in B16-OVA tumor bearing mouse models. In particular, NK^IL−12^ plus IL-2 treatment increased survival by 11.5 days, while NK^IL−12^ plus CTL and IL-2 prolonged survival for 16.5 days. Nevertheless, all mice eventually succumbed to the disease and no stable tumor regressions were reported (319).

CAR T cells have shown remarkable efficacy against hematologic malignancies but have yet to achieve clinical benefit against solid tumors. Engineering CAR-T cells to express IL-12 is under investigation to improve antitumor efficacy against solid tumors. In one study, i.v. infusion of IL-12 expressing anti-VEGFR2 CAR T cells induced curative regressions in 40–80% of mice in five different tumor models including 10–12-day-old B16F10 melanomas, MCA-205 sarcomas, MC38 colorectal adenocarcinomas, MC17-51 sarcomas or 12–14 days old CT26 colon adenocarcinomas (320). T cells singularly transduced with either anti-VEGFR-2 or IL-12 were markedly less effective (320). Because constitutive systemic expression of IL-12 can be toxic, inducible IL-12 expression strategies have been developed. By using an NFAT-responsive promoter, IL-12 could be expressed only after TCR engagement (320–322). This modification reduced toxicity but required lymphodepletion to achieve durable tumor regressions (320).

The efficacy of CAR T cell therapy in general is dependent upon lymphodepletion via chemotherapy or radiotherapy. In an attempt to eliminate the need for lymphodepleting preconditioning, CD19 CAR T cells expressing IL-12 (CD19/IL-12) have been investigated (323, 324). Indeed, treatment with CD19/IL-12 CAR T cells significantly enhanced the survival of CD19-EL4 tumor-bearing mice vs. treatment with CD19 CAR T cells (75 vs. 0% survival at day 60 after tumor inoculation) (323). Similarly, second generation CD19/IL-12 CAR T cells produced a 25% long-term survival rate in mice with established A20 B cell lymphomas. Without the IL-12 insert, all mice treated with CD19 CAR T cells succumbed to disease (324).

4H11-28z/IL-12 CAR T cells, which are specific for the MUC-16^ecto^ antigen and secrete IL-12, controlled SKOV3 human ovarian carcinomas in SCID-Beige mice following i.p. infusion 2 or 4 weeks after tumor implantation (325). Specifically, ~80 or 100% of mice treated 4 or 2 weeks, respectively, after tumor implantation with 4H11-28z/IL-12 CAR T cells survived for 90 days. In contrast, ~0% and 20% of mice treated 4 or 2 weeks, respectively, after tumor implantation with 4H11-28z CAR T cells, not containing the IL-12 gene, survived for 90 days (325).

Glypican-3 (GPC3)-specific CAR T cells with inducible expression of IL-12 (GPC3-28Z-NFAT-IL-12 CAR T) significantly inhibited or eliminated established PLC/PRF/5 and Huh-7 human HCCs (326). The IL-12 secreting CAR T cells resulted in decreased T_reg_ infiltration. Serum IFNγ and IL-12 levels were elevated although neither pathological changes to critical organs nor significant loss in body weight were observed (326). It should be noted, however, that these studies were performed in immunocompromised mice which is not a reliable model for immune-related adverse events.

#### Limitations

From a clinical standpoint, administrations of IL-12-transduced cells have been well-tolerated, with patients experiencing only transient symptoms of lymphopenia, fever, and malaise; however, significant, durable clinical responses have been elusive. From a manufacturing perspective, the transduction of allogeneic and xenogeneic cells is easier and more reproducible. However, these cells are rapidly recognized and rejected by the host immune system. Thankfully, recent advances in autologous cell transduction due to the emergence of CAR T cell immunotherapy has eliminated many concerns related to technical feasibility and patient heterogeneity (310).

### Sustained Release Platforms for Local Injection

Local administration of recombinant IL-12 protein is the most direct and most quantifiable strategy in terms of ensuring the accuracy and reproducibility of a delivered dose. However, recombinant cytokines disseminate rapidly from local injection sites into the systemic circulation (327). Thus, in order to maintain high levels of recombinant IL-12 in the tumor microenvironment while minimizing systemic exposure, sustained delivery technologies, capable of locally releasing proteins and cytokines over extended periods of time following direct injection, are under development (328).

#### Polymeric Microspheres

The encapsulation of pharmaceuticals in polymeric microspheres for sustained delivery has been explored for several decades (329, 330). The use of microspheres to deliver cytokines is a more recent trend. Synthetic polymers, such as poly-lactic co-glycolic acid (PLGA), poly-caprolactone (PCL), and poly-lactic acid (PLA), that have been widely explored for drug delivery, are being adapted to encapsulate and deliver various cytokines including IL-12 (55, 331–335). Human IL-12-loaded PCL:PLA microspheres, when co-delivered with human peripheral blood lymphocytes (PBLs), were shown to prevent engraftment of human tumors in SCID mice (333). PEG-IL-2 also suppressed tumor engraftment and growth, but not as effectively as IL-12-loaded microspheres. Although high levels of IL-12 and IFN-γ were found in sera after administration of IL-12-loaded microspheres, this localized delivery strategy was more effective at preventing tumor engraftment than repeated i.p. injections of IL-12 (336). Similarly, a single i.t. injection of IL-12-loaded PCL:PLA microspheres outperformed bolus injections of IL-12 in the inhibition of human head and neck tumor tissue xenografts containing human PBLs (337). IL-12-loaded microspheres completely suppressed engraftment in 50% of mice, whereas IL-12 alone slowed but could not eliminate tumor growth (337). In the above xenograft studies, antitumor activity was mediated by CD8^+^ T cells and CD56^+^ natural killer cells (333, 337). In yet another study, lung tumor xenografts using non-disrupted human lung tumor tissue were completely suppressed when injected with IL-12-loaded PLA microspheres 1 week after implantation (338). Mechanistically, quiescent CD4^+^ effector memory T cells present within the tumor microenvironment were reactivated upon exposure to high local levels of IL-12 (339). The proliferation of reactivated CD4^+^ cells and their production of IFN-γ facilitated tumor destruction (339).

In murine tumor models, i.t. injections of IL-12-loaded PLA microspheres eliminated up to 70% of Line-1 lung alveolar carcinomas (332). In contrast, i.t. injections of free IL-12 induced a complete response in only one of five treated mice. Interestingly, mice experiencing complete tumor regression following microsphere administration were more resistant to tumor rechallenge than mice vaccinated with either live or irradiated Line-1 cells co-injected with IL-12-loaded microspheres (332). In other studies, i.t. injections of IL-12-loaded PLA microspheres significantly reduced the incidence and number of spontaneous pulmonary tumor nodules (331, 340). When administered neoadjuvant to tumor resection, IL-12-loaded microspheres inhibited local recurrence, reduced pulmonary metastases, and improved disease-free survival as compared to resection alone (331). A similar study using more advanced Line-1 tumors, which were 1,000–1,300 mm^3^ at study onset, demonstrated that neoadjuvant i.t. injections of IL-12-loaded microspheres prevented both local recurrence and pulmonary metastases better than surgery alone (341). The addition of GM-CSF-loaded PLA microspheres enhanced antitumor activity and survival. However, more cytokines were not always better, as the addition of low dose IL-2 to neoadjuvant immunotherapy with IL-12- and GM-CSF-loaded microspheres abrogated antitumor immunity (341). A theme common to nearly all IL-12-based immunotherapies was that IL-12-loaded microspheres induced CD8^+^ T cell and NK cell activation, including increases in cytolytic function and IFN-γ expression. In-depth effector mechanisms are elegantly described in a series of papers by Egilmez and Kilinc et al. (69, 340, 342–345).

In addition to inducing a strong effector response, a potentially more important feature of i.t. injected IL-12-loaded microspheres is their potential to reverse immunosuppression in the tumor microenvironment. For example, a single i.t. injection of IL-12-loaded microspheres with and without GM-CSF-loaded microspheres induced apoptosis and elimination of tumor-infiltrating CD4^+^CD25^+^Foxp3^+^ T suppressor cells (69, 344). IL-12-loaded microspheres were also shown to reprogram immunosuppressive tumor-infiltrating macrophages (TIMs) and tumor-associated macrophages (TAMs) to a proinflammatory, antitumor phenotype (11, 346).

Microsphere platforms are well-suited to explore co-delivery of multiple cytokines. The combination of IL-12- and TNFα-loaded PLA microspheres was shown to be more effective than IL-12 and GM-CSF-loaded microspheres at eliminating MT-901 mammary carcinomas (347) and B16 melanomas (55). Nearly 70 and 40% of MT-901 tumor-bearing mice were rendered tumor-free following i.t. injections with dual cytokine loaded microspheres of IL-12/TNFα and IL-12/GM-CSF, respectively (347). Dual cytokine loaded microspheres of IL-12/TNFα also induced increases in tumor-specific cytokine release from effector cells as compared to microspheres containing either cytokine alone (334) or the combination of IL-12 and GM-CSF (55, 347).

A single i.t. injection of IL-12- and IL-18-loaded PLA microspheres outperformed injections of either cytokine-loaded microsphere alone in terms of delaying the growth of B16 melanomas (348). Unlike previous studies demonstrating the elimination of 70% of Line-1 tumors (332) and impressive reductions in metastatic lesions (331, 341), IL-12-loaded microspheres had no impact on either primary B16 tumor growth or pulmonary metastases (348). Interestingly, the triple combination of IL-18/IL-12/TNFα loaded in microspheres was found to reduce antitumor activity in the 4T1 model (335).

In her-2/neu transgenic mice, which develop spontaneous, multifocal mammary carcinomas, IL-12- and GM-CSF-loaded microspheres injected intratumorally caused complete regression of primary tumors in up to 40% of mice (349). Unfortunately, responses were temporary, and all tumors recurred. Interestingly, chronic immunotherapy with cytokine-loaded microspheres could not control tumor burden due to a progressive decline in the intensity of antitumor T cells and an increase in tumor-infiltrating CD4^+^CD25^+^Foxp3^+^ T suppressor cells with repeated injections (349, 350). Subsequent studies have shown that administration of cyclophosphamide can block the post-immunotherapy increase in T suppressor cells (351, 352).

Recently, intratumoral delivery of IL-12 microspheres (IL-12 MS) in combination with stereotactic body radiation (SBRT) resulted in remarkable tumor regression in several types of preclinical pancreatic ductal adenocarcinoma mouse models (353). This study showed that intratumoral levels of IFN-γ were enhanced following the treatment of SBRT/IL-12 MS, which in turn increased CD8^+^ T cell activation. SBRT/IL-12 MS treatments also established systemic tumor immunity that was confirmed by antitumor effects on liver metastases (353).

#### Chitosan and poly-N-acetyl glucosamine

Chitosan (CS) is a bioadhesive polysaccharide derived primarily from the exoskeletons of crustaceans. It is non-toxic, biodegradable, and non-immunoreactive (354) with an established safety profile in humans. Co-formulation of IL-12 in CS (CS/IL-12) has been shown to increase the i.t. residence of IL-12 from 1–2 days to 1–2 weeks (23). CS is believed to hinder IL-12 diffusion through viscous and electrostatic interactions.

The sustained presence of CS/IL-12 induced complete regression of 80–100% of mice bearing MC38, Panc02, CT26, E0771, and MB49 tumors (23, 355). Administration of CS/IL-12 neoadjuvant to 4T1 tumor resection resulted in complete clearance of lung metastases and long-term survival in about two-thirds of mice. In contrast, all mice treated with surgery alone died within 38 days and mice receiving IL-12 without CS neoadjuvant to resection had a median survival of 46 days (52). Cured mice rejected a subsequent challenge with live 4T1 cells, but not live Renca cells illustrating the specificity of the antitumor immune response. In another demonstration of systemic tumor-specific immunity from localized IL-12, 70% of mice bearing both flank and orthotopic MB49 bladder tumors were able to clear the flank tumor following intravesical administration of CS/IL-12 (53). In contrast, mice with only a flank tumor, did not benefit from intravesical CS/IL-12 in a naïve bladder. These data indicate that intravesical CS/IL-12 induced systemic tumor-specific immunity only when IL-12 was localized in or near a tumor. In a subsequent study, orthotopic bladder tumor regression following intravesical CS/IL-12 immunotherapy was associated with a rapid infiltration of macrophages and granulocytes followed by increases in CD4^+^ and CD8^+^ effector T cells along with a reduction of immunosuppressive T_regs_ and MDSCs (14). Importantly, local administrations of CS/IL-12 did not result in significant toxicity, such as elevated liver enzymes, commonly observed following regular, free IL-12 injections (52).

A polysaccharide gel matrix, designated F2 gel, is comprised of 70% deacetylated poly-N-acetyl glucosamine (p-GlcNAc) co-formulated with lactate salt. p-GlcNAc gels were evaluated as slow-release cytokine depots for GM-CSF, IL-2, and IL-12 (356–360). In particular, i.t. injections of IL-2 in p-GlcNAc gel delayed the growth of OVA-transfected AE17 malignant mesotheliomas (359). Interestingly, GM-CSF and IL-12 depots had no impact on mesothelioma growth, although only a single i.t. injection was given. The p-GlcNAc gel by itself induced a strong, transient inflammatory response which was thought to be due to a foreign body reaction or toxic species that may have been contained within the gel (361, 362).

#### Liposomes

Human IL-12 has been encapsulated in multilamellar liposomes for sustained release after i.t. injection (363). Antitumor activity on xenografts of human lung tissues indicated that liposomal encapsulation is a promising approach capable of eliminating tumor cells and inducing lymphocyte infiltration 2 weeks after i.t. injection. Liposomal leakage and/or IL-12 liposome dissemination could be problematic following delivery of large doses (10 μg/mouse) as significant sustained levels of IL-12 and IFN-γ were found in sera of treated mice (363).

Capitalizing on the tumor disruption caused by cationic liposomes, IL-12- and TLR4 ligand monophosphoryl lipid A (MPL)-loaded cationic liposomes were injected i.t. to treat 4T1 tumors. This combination produced an abscopal effect which arrested growth in both treated and distal tumors but did not result in tumor elimination (364).

#### Limitations

To date, sustained release technologies for recombinant IL-12 have not been evaluated in clinical studies. While sustained release technologies are designed to maximize IL-12 concentrations in the tumor microenvironment, some amount of delivered IL-12 can be expected to reach the systemic circulation. However, due to the infrequency of administration for these long-acting platforms, they are unlikely to induce the same lethal toxicities that were observed following the frequent systemic IL-12 administrations in early clinical trials (42). Nevertheless, it will be essential to perform rigorous pharmacokinetic and toxicology studies prior to clinical translation of any sustained recombinant IL-12 platform.

A possible limitation of the microsphere encapsulation technology is the use of organic solvents during the encapsulation process. Organic solvents can denature cytokines immediately or adversely affect long term storage. One study indicated that cytokines lose about half of their bioactivity during microencapsulation (365). Loss of bioactivity appears to be cytokine dependent (366). Specific activity of IL-12 after encapsulation in PLA microspheres was 90%. However, more than 80% of the bioactivity of IL-12 was lost when PLA/IL-12 microspheres were stored for 8 days (366).

In addition, because many commonly used biodegradable polymers are comprise of acidic monomers, polymer degradation often forms highly acidic microenvironments that may inactivate cytokines (188). A number of techniques are under investigation to either raise pH (367) or stabilize encapsulated cytokines (368).

A common limitation of liposomal carriers is their inherent leakiness, and therefore the choice of lipid and modifications to stabilize the bilayer are critical for sustained release. High temperature phase-transition lipids, such as DSPC, increase the retention of cargo and enhance drug circulation time. Incorporation of cholesterol into the lipid bilayer increases the rigidity and further decreases leakage of interior cargo (369). Another major limitation of liposomes is opsonization by serum proteins and clearance by phagocytes of the reticuloendothelial system (RES). PEGylation can reduced RES clearance, however, PEG can sterically inhibit ligand-cell interaction (370). If administered i.t., loss of tumor targeting is likely not detrimental.

### Future Strategies

While some localized IL-12 immunotherapies have shown limited efficacy in clinical studies, a long-awaited breakthrough leading to widespread application of localized IL-12 as a monotherapy does not appear imminent. However, there are a number of near-term opportunities to combine localized IL-12 with traditional or experimental cancer management approaches. And in fact, most contemporary experimental therapies for aggressive or advanced cancers involve combination approaches. Thus, near future opportunities involving translatable combination approaches are explored in the following sections.

#### Neoadjuvant to Resection

The majority of cancer patients with solid tumors undergo tumor resection as part of their treatment. Surgery, by itself, is unable to prevent recurrence and/or metastasis which is responsible for 9 out of 10 cancer-related deaths. Patients at high risk of recurrence receive adjuvant chemotherapy and/or radiotherapy following resection. While chemotherapy and radiotherapy may help reduce the risk of recurrence, they induce life-altering side effects and fail in a significant fraction of patients. As a result, most metastatic cancers arise from previously treated non-metastatic disease (371–373).

Administration of localized IL-12 to the tumor microenvironment prior to surgery has the potential to reduce recurrence rates and/or eliminate occult metastases through the induction of systemic, tumor-specific immunity. As mentioned above, our own investigation demonstrated that neoadjuvant i.t. injections of CS/IL-12 prior to resection can improve overall survival from 0 to 65% in a highly metastatic 4T1 breast carcinoma model (52). Cured mice demonstrated tumor-specific immunity via tumor rechallenge, CTL activity, and ELISPOT assays.

If proven to be safe, localized IL-12 can be administered neoadjuvant to any solid tumor resection, not just in high risk patients. In fact, at the time of surgery, it is likely that a significant fraction of patients already has occult metastases that cannot be detected via radiographic imaging. The median primary breast tumor size for which 100% of patients had micrometastases was found to be only 1.8 cm (374).

While most immune-oncology trials focus on developing therapies for metastatic disease, an approach, such as neoadjuvant localized IL-12, capable of preventing metastasis in the first place, may be a more effective strategy to reducing cancer mortality.

#### Adjuvant to Chemo

The combination of IL-12-based immunotherapy and chemotherapy (“biochemotherapy”) is also under exploration. Several clinical studies have shown significant clinical benefit when certain cytotoxic drugs are combined with IL-12 (181, 375, 376). There are three distinct advantages for combining chemotherapy with IL-12 immunotherapy. First, chemotherapy provides a debulking benefit with the elimination of some or most of the tumor. Second, some chemotherapeutics can deplete immunosuppressive cells. Cyclophosphamide has been shown to deplete T_regs_ while 5-fluorouricil and gemcitabine have been shown to deplete MDSCs. Third, some chemotherapies cause upregulation of T cell-attracting chemokines in the tumor (377). For example, melanoma-bearing mice treated with temozolomide, cisplatin, or dacarbazine increase expression of CCL5, CXCL9, and CXCL10 (378).

The timing of chemotherapy relative to local IL-12 immunotherapy is expected to be critical. Unlike the neoadjuvant resection approach mentioned above, chemotherapy should be administered prior to IL-12 therapy to avoid elimination of beneficial proliferating immune cells. Chemotherapy may synergize with IL-12 by reducing tumor burden and creating a source of tumor antigen for *in situ* vaccination. In fact, certain chemotherapies can induce an immunogenic cell death that is capable of priming an antitumor immune response (379).

To date, several examples demonstrate the promise of chemotherapy plus local IL-12 delivery. Antitumor efficacy of pIL-12/PPC is improved with paclitaxel, cyclophosphamide, or Taxol/Paraplatin (172, 175, 177). The antitumor effect of IL-12 lipoplexes against intracranial glioma was substantially improved when combined with FDA-approved carmustine (BCNU) wafers (176). Finally, a Phase-1 clinical trial found that intraperitoneal IL-12 gene therapy in combination with intravenous pegylated liposomal doxorubicin resulted in greater clinical benefit relative to the chemotherapeutic alone (57.1% vs. 40–45.3%) in patients (*n* = 11) with platinum-resistant epithelial ovarian cancer (181).

#### Adjuvant to Radiation

Standard-of-care radiotherapy of inoperable tumors can be administered prior to local IL-12 delivery. Radiation-induced tumor cell death can supply a source of tumor antigens, while radiation has been shown to upregulate co-stimulatory molecules on the surface of tumor cells which makes them more susceptible to immune-mediated killing (380). The addition of tumor irradiation after pIL-12+EP was found to improve antitumor activity (130). Similarly, radiotherapy improved the antitumor activity of i.t. oncolytic adenoviruses expressing IL-12 and GM-CSF (381). It has also been found that radiotherapy can induce infiltration of MSCs in the tumor site. Through a combination of radiation and IL-12-expressing MSCs, both primary tumor growth and metastases were reduced in HCa-I and Hepa 1-6 hepatoma models (382). In another study, radiation along with an i.t. injection of an IL-12-encoding adenoviral vector greatly reduced tumor growth and metastasis compared to either treatment alone in a BNL-P2 hepatocellular carcinoma model, while a near-complete abscopal response occurred in a CT26 colorectal cancer model (383). An abscopal response was also induced in a fully humanized rhabdomyosarcoma xenograft model. Mice with bilateral tumors were treated with a combination of single-tumor radiation and systemic NHS-IL12, which resulted in significantly lower tumor burdens than either individual treatment (114).

#### Adjuvant to Ablation

Cryoablation, radiofrequency ablation (RFA), microwave ablation (MWA), embolic microspheres and high intensity focused ultrasound (HIFU) are routinely used in the clinic to destroy some operable and many inoperable solid tumors. Irreversible electroporation is another ablative technology in clinical and preclinical development (384–387). All of these strategies have the potential to induce *in situ* vaccinations through the release of tumor antigen, although abscopal responses or robust anti-tumor immune responses are rare. Among the ablation strategies, cryoablation appears distinct from most other ablation approaches that kill via high temperatures and result in coagulation, enzyme inactivation, and antigen denaturation. Cryoablation is thought to mostly preserve tumor antigen structure (388, 389) and has been shown to induce greater levels of tumor antigen uptake by dendritic cells compared to RFA (390). In addition, cryoablation also induces higher levels of inflammatory cytokines, such as IL 1, IL 6, and TNFα, compared to RFA and MWA (391, 392). Perhaps most importantly, cryoablation was recently shown to induce expansion of oligoclonal T cells both in tumor tissues and in peripheral blood of kidney cancer patients (393).

The addition of localized IL-12 to the above ablation strategies may enhance tumor control as well as antitumor immunity. Our recent preclinical data, demonstrated that the combination of cryoablation and CS/IL-12 could eliminate multiple tumor types and induce abscopal immunity in a bilateral flank model (394). Ongoing studies are aimed at determining mechanisms of systemic tumor control.

#### Combinations With Immune Checkpoint Blockade

Immune checkpoint inhibitors are ineffective against non-inflamed, “cold” tumors. Localized IL-12 induces profound intratumoral infiltration that can be expected to synergize with checkpoint inhibitors. Indeed, the addition of anti-PD-1 or anti-CTLA-4 improved the antitumor activity of L19-IL-12 in multiple murine tumor models (395). Similarly, systemic anti-CTLA-4 in combination with intratumoral IL-12 induced synergistic antitumor responses in GL-26 glioblastoma and SMA-560 astrocytoma models (396). Also, as mentioned in a previous section, i.t. injections of VV-IL-12 and VV-IL7 enhanced the responsiveness of poorly immunogenic tumors to checkpoint inhibition (268). Finally, a recent Phase II study of i.t. pIL-12+EP and systemic pembrolizumab in patients with non-infiltrated melanomas resulted in a 41% objective response rate with 36% complete responses (397). Correlative studies demonstrated increased antigen cross-presentation along with enhance T cell infiltration and activity leading to higher than expected clinical responses (397).

## Conclusions

IL-12 enhances cytotoxic T and NK cell activity while reversing tumor-induced immunosuppression, inhibiting angiogenesis, increasing lymphocyte trafficking and antigen presentation either directly or through induction of IFNγ (398). Based on these diverse mechanisms as well as its profound antitumor activity in numerous preclinical studies, IL-12 is arguably one of, if not, the most potent antitumor cytokine evaluated to date. Unfortunately, the much-anticipated clinical translation of IL-12-based immunotherapies suffered a tremendous setback in the late 1990s/early 2000s due to severe clinical toxicities associated with systemic IL-12 injections.

Through the development of several clever approaches to localize IL-12 to the tumor microenvironment while limiting systemic exposure, IL-12-based immunotherapies are making a comeback. Several clinical studies evaluating localized IL-12 have been initiated (Table 2) with more on the way. While each delivery strategy has limitations, the approaches reviewed above may retain enough IL-12 in the tumor while eliminating the need for frequent systemic injections. By reducing the potential for IL-12-mediated toxicities associated with systemic injections, localized IL-12 can expand the therapeutic window and finally allow IL-12 to fulfill its considerable potential.

As exemplified by the current immune-oncology clinical trial landscape, combination approaches appear to be most effective for accelerating clinical impact. Several promising preclinical combination approaches with localized IL-12 are likely to be translated in the near future. There is unprecedented interest in finding immunomodulators that can enhance lymphocytic infiltration in order to improve the efficacy of checkpoint inhibitors. Localized IL-12, based on its ability to drive Th1 responses, enhance cytolytic activity, and protect T cells from PD-1/PD-L1 exhaustion and IFNγ-induced apoptosis may be an ideal partner for checkpoint inhibitors.

If safety concerns are assuaged, localized IL-12 could be used in earlier stage cancer patients as a neoadjuvant to resection. For tumors that are inoperable, combining localized IL-12 with ablation may help increase local as well as distant tumor control. With the interesting combination approaches, as well as the uptick in IL-12-based immunotherapies in clinical trials, there is reason to believe that localized IL-12 may play a major role in cancer immunotherapy in the near future.

## Author Contributions

KN, MV, SM, JH, EW, TG, and DZ researched and contributed various sections of the manuscript. KN and DZ outlined and revised the manuscript. All authors contributed to the article and approved the submitted version.

## Conflict of Interest

The authors declare that the research was conducted in the absence of any commercial or financial relationships that could be construed as a potential conflict of interest.

## Abbreviations

A-NK^IL−12^ or activated NK^IL−12^: Activated primary natural killer (NK) cells transduced to express IL-12
AdCMVIL-12 or AdCMVmIL-12: Recombinant defective adenovirus expressing IL-12 or mIL-12 under control of a CMV promoter
AdmIL-12.1: Adenoviral vector expressing murinedIL-12 protein under the control of the HCMV promoter and SV40 polyadenylation sequences
Ad-DHscIL12: Adenovirus with replication dependent on hypoxia-inducible factor (HIF) activity expressing single-chain IL-12
Ad-IL-12 or AdIL-12 or Ad.IL-12 or AdmIL-12 or Ad.mIL-12: Adenoviral vectors encoding human or murine interleukin-12
Ad-RTS-IL-12: Inducible adenoviral vector engineered to express IL-12 under the control of the RheoSwitch Therapeutic System® (RTS) gene switch
AdTCPmIL-12: Recombinant replication-defective adenoviral vector expressing murine interleukin-12 (mIL-12), driven by a modified CALC-I promoter (TCP)
Ad-TD-IL-12: Oncolytic triple-deletion (TD) adenoviral vector encoding wild-type IL-12
Ad-TD-nsIL-12: Oncolytic triple-deletion (TD) adenoviral vector encoding non-secreting IL-12
ADCC: Antibody-Dependent Cellular Cytotoxicity
Ad.scIL-12: Adenoviral vector expressing single-chain IL-12
Ad5-yCD/mutTKSR39rep-hIL12: A replication-competent oncolytic adenovirus encoding the murine pro-inflammatory cytokine interleukin-12 (IL-12) gene and two suicide fusion genes, a yeast cytosine deaminase (yCD) and a mutant form of herpes simplex virus type 1 thymidine kinase (HSV-1 TKSR39)
Ad.5/3.cRGD-mIL12p70: A replication-deficient double targeted Ad5 backbone-based vector carrying a chimeric Ad5/3 fiber with integrin-binding RGD motif incorporated in its Ad3 knob domain expressing murine IL-12p70
Ad/scIL-12-B7TM: Adenoviral vector encoding membrane-anchored murine single-chain IL-12 with B7-1 transmembrane and cytoplasmic domains
AIDS: Acquired Immune Deficiency Syndrome
ATRA-cationic liposome/IL-12 pDNA: pIL-12 complexed with cationic liposomes incorporating all-trans-retinoic acid
ALVAC-IL12: Recombinant canarypox virus encoding IL-12
CAR: Chimeric Antigen Receptor
CBD: Collagen Binding Domain
CBD-IL-12: Collagen-binding immunocytokine comprised of A3 CBD of von Willebrand Factor fused to both subunits of IL-12
CD19/IL-12: CD19 CAR T cells expressing IL-12
CEA: Carcinoembryonic Antigen
chTNT-3/huIL-12: Necrosis-targeting immunocytokine comprised of huIL-12 fused to the variable heavy chain of chTNT-3 antibody
CTL: Cytotoxic T Lymphocytes
DC-AdCMVIL-12: Dendritic cells transfected with adenovirus expressing IL-12 under control of the CMV promoter
DNA: Deoxyribonucleic Acid
ELISPOT: Enzyme-Linked Immune Absorbent Spot
FDA: Food and Drug Administration
GM-CSF: Granulocyte-Macrophage Colony-Stimulating Factor
GPC3-28Z-NFAT-IL-12 T cells: Glypican-3-specific CAR T cells with NFAT-inducible expression of IL-12
HLA: Human Leukocyte Antigen
HSV-IL-12: Herpes simplex viruses (HSV) encoding Il-12
huBC1-IL12: Immunocytokine comprised of two molecules of IL-12 fused to each of the IgG heavy chains of humanized BC-1 antibody
Hu-KS-IL-12: Immunocytokine comprised of IL-12 fused to the Fc fragment of humanized KS antibody
Hu-14.18-IL-12: Immunocytokine comprised of IL-12 fused to GD2 targeting hu14.18 antibody
IFN: Interferon
IFNAR: Type I Interferon Receptor
DMP/IL-12: pIL-12 complexed with DMP cationic micelles
DMRIE/DOPE/pIL-12: pIL-12 complexed with N-(1-(2,3-dimyristyloxypropyl)-N,N-dimethyl-(2-hydroxyethyl)ammonium bromide/dioleoyl phosphatidylethanolamine (DMRIE/DOPE) cationic lipids
IL-12: Interleukin-12
IL-12M: Modified IL-12; an N-glycosylation mutant of IL-12 at Asn220
IL12-IL2: Immunocytokine comprised of IL-12 and IL-2 fused to anti-CD30 scFv antibody
IL-12-L19: Immunocytokine comprised of IL-12 fused to L19 scFv antibody
IL12-MSA: IL-12 fused to mouse serum albumin (MSA)
IL12-MSA-Lumican: IL-12 fused to mouse serum albumin (MSA) and lumican, a collagen-binding proteoglycan
IL-12/PVP: IL-12 complexed with polyvinylpyrridilone (PVP), a cationic polymer
IL12-SS1 (Fv): Immunocytokine comprised of p35 subunit of single-chain IL-12 fused to SS1, a mesothelin-binding single-chain variable fragment (scFv)
Lenti-mIL-12-MSCs: Bone-marrow derived MSCs transfected with lentivirus to express IL-12
MC/pmIL-12: pmIL-12 complexed with mannosylated chitosan (MC)
MDSCs: Myeloid-Derived Suppressor Cells
MeVac FmIL-12: Attenuated measles viruses (MeVac) encoding IL-12
MHC: Major Histocompatibility Complex
MoMLV-mIL-12: Moloney murine leukemia virus (MoMLV) expressing IL-12
mPEG/pmIL-12: Polymersomes comprised of pmIL-12 complexed with polyphosphazene particles modified with DPA and mPEG
MSCs: Mesenchymal Stem Cells
mscIL-12.her2.IgG3: Mouse single-chain IL-12 fused to an anti-HER2/neu IgG3 antibody
MSC/IL-12 or MSC-IL-12: Mesenchymal stem cells (MSCs) expressing IL-12
L19-mIL12: Immunocytokine comprised of murine IL-12 fused to L19 scFv antibody
NHS-IL12: Necrosis-targeting immunocytokine comprised of two single-chain IL-12 molecules fused to full-length NHS76 antibody
NHS-muIL12: Murine analog of NHS-IL12
NK cells: Natural Killer cells
oHSV-IL-12: Oncolytic herpes simplex viruses (oHSV) encoding IL-12
PAGA/pmIL-12: pmIL-12 complexed with poly-a-(4-aminobutyl)-L-glycolic acid (PAGA), a biodegradable polyester
PBL: Peripheral Blood Lymphocytes
PD-1: Programmed Cell Death Protein 1
PD-L1: Programmed Death-Ligand 1
PEI:IL-12: IL-12 complexed with polyethyleneimine (PEI)
pIL-12: Plasmid DNA encoding IL-12
pIL-12/PPC: pIL-12 complexed with polyethyleneglycol-polyethylenimine-cholesterol lipopolymer
pmIL-12: Plasmid DNA encoding murine IL-12
pIL-12+EP: Intratumoral injection of pIL-12 immediately followed by electroporation
PRX/pIL-12: pIL-12 complexed with polytraxane
pSFV10-E-IL12: Enhanced SFV vector with 10x higher gene expression of recombinant IL-12 protein
rAd/IL-12M: Recombinant adenovirus expressing IL-12M
rNDV-anti-CD28-mIL-12: Newcastle disease virus (NDV) expressing immunocytokine comprised of anti-CD28 antibody fused to mIL-12
rNDV-anti-PDL1-mIL-12: Newcastle disease virus (NDV) expressing immunocytokine comprised of anti-PDL1 antibody fused to mIL-12
rSFV/IL12: Recombinant Semliki Forest Viruses (SFV) encoding IL-12
rVSV-IL12: Recombinant Vesicular stomatitis virus (VSV) encoding IL-12
rVV-mIL-12: Recombinant Vaccinia virus (VV) encoding murine IL-12
SCID: Severe Combined Immunodeficient
scIL-12: Single-chain IL-12
SFV-IL12: Semliki Forest Viruses encoding IL-12
SFV-IL-12: Semliki Forest Viruses encoding IL-12
STING: Stimulator of Interferon Genes
TAMs: Tumor Associated Macrophages
TLR: Toll-like Receptor
TNF: Tumor Necrosis Factor
UCB-MSC-IL12M: Human umbilical cord blood-derived mesenchymal stem cells (UCB-MSCs) expressing IL-12M
VV-IL-12: Vaccinia virus (VV) expressing IL-12
WSLP/p2CMVmIL-12: IL-12 expression plasmid complexed with water-soluble lipopolymers
4H11-28z/IL-12: CAR T cells which are specific for the MUC-16ecto antigen and secrete IL-12
6B11ScFv-mIL-12: Immunocytokine comprised of mIL-12 fused to scFv of 6B11 antibody
9L-IL12: 9L gliosarcoma cells engineered to express IL-12.
